# *Lachancea fermentati* Strains Isolated From Kombucha: Fundamental Insights, and Practical Application in Low Alcohol Beer Brewing

**DOI:** 10.3389/fmicb.2020.00764

**Published:** 2020-04-23

**Authors:** Konstantin Bellut, Kristoffer Krogerus, Elke K. Arendt

**Affiliations:** ^1^School of Food and Nutritional Sciences, University College Cork, Cork, Ireland; ^2^VTT Technical Research Centre of Finland Ltd., Espoo, Finland; ^3^APC Microbiome Ireland, University College Cork, Cork, Ireland

**Keywords:** brewing, fermentation, kombucha, *Lachancea fermentati*, lactic acid, NABLAB, response surface methodology, whole genome sequencing

## Abstract

With a growing interest in non-alcoholic and low alcohol beer (NABLAB), researchers are looking into non-conventional yeasts to harness their special metabolic traits for their production. One of the investigated species is *Lachancea fermentati*, which possesses the uncommon ability to produce significant amounts of lactic acid during alcoholic fermentation, resulting in the accumulation of lactic acid while exhibiting reduced ethanol production. In this study, four *Lachancea fermentati* strains isolated from individual kombucha cultures were investigated. Whole genome sequencing was performed, and the strains were characterized for important brewing characteristics (e.g., sugar utilization) and sensitivities toward stress factors. A screening in wort extract was performed to elucidate strain-dependent differences, followed by fermentation optimization to enhance lactic acid production. Finally, a low alcohol beer was produced at 60 L pilot-scale. The genomes of the kombucha isolates were diverse and could be separated into two phylogenetic groups, which were related to their geographical origin. Compared to a *Saccharomyces cerevisiae* brewers’ yeast, the strains’ sensitivities to alcohol and acidic conditions were low, while their sensitivities toward osmotic stress were higher. In the screening, lactic acid production showed significant, strain-dependent differences. Fermentation optimization by means of response surface methodology (RSM) revealed an increased lactic acid production at a low pitching rate, high fermentation temperature, and high extract content. It was shown that a high initial glucose concentration led to the highest lactic acid production (max. 18.0 mM). The data indicated that simultaneous lactic acid production and ethanol production occurred as long as glucose was present. When glucose was depleted and/or lactic acid concentrations were high, the production shifted toward the ethanol pathway as the sole pathway. A low alcohol beer (<1.3% ABV) was produced at 60 L pilot-scale by means of stopped fermentation. The beer exhibited a balanced ratio of sweetness from residual sugars and acidity from the lactic acid produced (13.6 mM). However, due to the stopped fermentation, high levels of diacetyl were present, which could necessitate further process intervention to reduce concentrations to acceptable levels.

## Introduction

Humans have utilized yeasts for the preparation of their foods and beverages long before they even knew of their existence, and beer brewing has been a human activity ever since the Neolithic period (Meussdoerffer, 2009). But it was not until the introduction of brewing with pure culture yeast by Emil Christian Hansen that brewers started to consciously select yeasts for specific purposes (Meussdoerffer, 2009). The species *Saccharomyces cerevisiae* especially, has been harnessed as a trustworthy workhorse in the production of beer, and production volumes have been growing to almost two billion hectoliters in 2018 (Barth-Haas Group, 2019).

However, emerging lifestyle trends, demographics and stricter legislation have led to a slowdown in beer volume growth over the past years, while the non-alcoholic and low alcohol beer (NABLAB) sector has seen a strong and steady growth, which is forecast to continue (Bellut and Arendt, 2019). There are two fundamentally different approaches when it comes to NABLAB production: physical dealcoholization by means of thermal or membrane methods to remove the ethanol after its formation (Müller et al., 2017), and biological methods like stopped fermentation to limit ethanol production in the first place (Brányik et al., 2012).

Another old, biological method for NABLAB production has seen a revival in recent years: the application of non-*Saccharomyces* yeasts (also called non-conventional yeasts) with limited ability to ferment wort sugars, resulting in a low ethanol production. This method was already mentioned in 1929 (Glaubitz and Haehn, 1929), and the proposed species, *Saccharomycodes ludwigii*, has been investigated thoroughly (Narziß et al., 1992; Liu et al., 2011; Mohammadi et al., 2011; Meier-Dörnberg and Hutzler, 2014; Mortazavian et al., 2014; Saerens and Swiegers, 2014; De Francesco et al., 2015, 2018; Jiang et al., 2017; Bellut et al., 2018). However recently, research into other non-*Saccharomyces* species to produce NABLAB has gained momentum (Bellut and Arendt, 2019). Researchers have been looking into isolating yeasts from non-cereal environments, to take advantage of their inability to consume the most abundant wort sugars maltose and maltotriose. Such environments include, for example, grapes and wine (Saerens and Swiegers, 2014; Estela-Escalante et al., 2016), honey (De Francesco et al., 2015), glaciers in Italy and the Antarctica (Thomas-Hall et al., 2010; De Francesco et al., 2018), Japanese miso (Sohrabvandi et al., 2009; Mohammadi et al., 2011) and, more recently, kombucha (Bellut et al., 2018, 2019a).

To date, more than 27 yeast genera have been found in kombucha cultures with up to 25 different species inhabiting a single culture (Jayabalan et al., 2014; Marsh et al., 2014; Reva et al., 2015; Chakravorty et al., 2016). One of the yeast genera associated with kombucha fermentation is the *Lachancea* genus, among which, *Lachancea fermentati* was first recorded by Marsh et al. (2014), and has since been reported to be the most abundant *Lachancea* species in kombucha (Chakravorty et al., 2016). *L. fermentati* has mostly been associated with grape must and kefir (Porter et al., 2019b) but the species was recently proposed as a novel brewing species to create sour beer or low alcohol beer (Osburn et al., 2018; Bellut et al., 2019a). The proposed applications are motivated by the fact that strains of the genus possess the uncommon ability to produce significant amounts of lactic acid during alcoholic fermentation. The production of high amounts of lactic acid by yeasts is an underexplored trait of both the *Lachancea* and *Saccharomyces* genus (Sauer et al., 2010; Jolly et al., 2014; Hranilovic et al., 2017). Lactic acid production is facilitated by the enzyme lactic acid dehydrogenase (LDH), which catalyzes the formation of lactic acid from pyruvate, the product of glycolysis. From a metabolic view-point, this pathway is an alternative, simultaneous means of NADH recycling to NAD^+^, with the more common pathway in yeast being via the production of ethanol (Sauer et al., 2010).

Lactic acid production in *Lachancea fermentati* has received little attention, but has been associated with *Lachancea thermotolerans*, where it has been shown to be highly strain-dependent (Porter et al., 2019b). Osburn et al. (2018) proposed the use of lactic acid-producing species like *Lachancea fermentati* to produce single-culture sour beer, making the use of lactic acid bacteria for souring redundant. Bellut et al. (2019a) proposed the use of *Lachancea fermentati* to produce low alcohol beer by stopping fermentation of a diluted wort and exploiting its lactic acid production to counteract residual wort sweetness.

In this study, we investigated four *Lachancea fermentati* strains isolated from four individual kombucha cultures. To better understand the variation in these four strains, whole-genome sequencing of the isolates and the CBS 707 type strain was performed. The strains were characterized for important brewing characteristics like sugar consumption, flocculation behavior, and susceptibility to stress factors like ethanol, low pH and high osmotic pressure. A screening in wort fermentations was performed to show differences in lactic acid production, sugar consumption and the production of volatile fermentation by-products. Further investigation involved an assessment of fermentation conditions and their impact on lactic acid production. Fermentation parameters studied were wort extract, fermentation temperature, and pitching rate, and results were evaluated via response surface methodology (RSM). Alterations of the sugar profile was investigated as another tool to enhance lactic acid production in wort. Finally, a low alcohol beer (<1.3% ABV) was produced by stopped fermentation at 60 L pilot scale and sensory evaluation was conducted with a trained panel.

## Materials and Methods

### Yeast Strains

The *Lachancea fermentati* strains KBI 1.2, KBI 3.2, KBI 5.3, and KBI 12.1 (Table 1) were isolated from four individual kombucha cultures according to Bellut et al. (2019a). CBS 707, the *Lachancea fermentati* type strain, was sourced from the CBS collection (Westerdijk Fungal Biodiversity Institute, Utrecht, Netherlands). The brewers’ yeast WLP001 (California Ale Yeast) was sourced from White Labs (San Diego, CA, United States).

**TABLE 1:** The ploidy and amount of homozygous and heterozygous single nucleotide polymorphisms (SNPs) observed in the *Lachancea fermentati* strains in comparison to the CBS 6772 reference genome.

**Strain name**	**Origin**	**Measured ploidy**	**Homozygous SNPs**	**Heterozygous SNPs**	**Total SNPs**
CBS 6772	Spoiled strawberry soft-drink, South Korea	–	–	–	–
CBS 707	Sediment of peppermint, Unknown	2	20281	838	21119
KBI 1.2	Kombucha, USA (Florida)	2	43937	237929	281866
KBI 3.2	Kombucha, USA (Arizona)	2	44797	235170	279967
KBI 5.3	Kombucha, Australia	1	21245	965	22210
KBI 12.1	Kombucha, USA (Hawaii)	2	45237	205790	251027

### Genomics

#### DNA Content by Flow Cytometry

Flow cytometry was used to estimate the ploidy of the yeast strains essentially as described by Haase and Reed (2002). Cells were grown overnight in YPD medium (1% yeast extract, 2% peptone, 2% glucose), and approximately 1 × 10^7^ cells were washed with 1 mL of 50 mM citrate buffer. Cells were fixed with ice-cold 70% ethanol, and incubated overnight at −20°C. Cells were then washed with 50 mM citrate buffer (pH 7.2), resuspended in 50 mM citrate buffer containing 0.25 mg/mL RNAse A and incubated overnight at 37°C. Proteinase K was then added to a concentration of 1 mg/mL, and cells were incubated for 1 h at 50°C. Cells were then stained with SYTOX Green (2 μM; Life Technologies, United States), and their DNA content was determined using a FACSAria IIu cytometer (Becton Dickinson, United States). DNA contents were estimated by comparing fluorescence intensities. In addition to the *L. fermentati* strains, analysis was also performed on *S. cerevisiae* haploid (CEN.PK113-1A) and diploid (CEN.PK) reference strains. Measurements were performed on duplicate independent yeast cultures, and 100,000 events were collected per sample during flow cytometry.

#### DNA Extraction and Sequencing

DNA was extracted from pellets using the Sigma GenElute Bacterial Genomic DNA Kit (Sigma-Aldrich, St. Louis MO, United States). After DNA isolation, DNA was quantified using the Qubit High Sensitivity DNA assay (Thermo Fisher Scientific, Waltham, MA, United States) and shotgun metagenomic libraries were prepared using the Nextera XT library preparation kit (Illumina, San Diego, CA, United States) as described by the manufacturer. The final libraries were sequenced on an Illumina NextSeq using a 300 cycle V2 Mid-Output kit as per Illumina guidelines. The raw sequencing reads generated in this study have been submitted to NCBI-SRA under BioProject number PRJNA587400 in the NCBI BioProject database.

#### Bioinformatics

The 150 bp paired-end reads were quality-analyzed with FastQC (Andrews, 2010) and trimmed and filtered with Trimmomatic (Bolger et al., 2014). Reads were aligned to a reference genome of *L. fermentati* CBS 6772 (NCBI Accession GCA_900074765.1) using SpeedSeq (Chiang et al., 2015). Variant analysis was performed on aligned reads using FreeBayes (Garrison and Marth, 2012). Prior to variant analysis, alignments were filtered to a minimum MAPQ of 50 with SAMtools (Li et al., 2009). The median coverage over 1000 bp windows was calculated with mosdepth (Pedersen and Quinlan, 2018) and visualized in R.

In addition, to test if any of the strains were interspecies hybrids, the trimmed reads were also aligned to a concatenated reference genome consisting of the assembled genomes of the twelve *Lachancea* species available at GRYC^1^. The median coverage over 1000 bp windows was again calculated with mosdepth, and was visualized in R using modified scripts from sppIDer (Langdon et al., 2018).

For phylogenetic analysis, consensus genotypes of the *L. fermentati* strains were called from the identified variants using BCFtools (Li, 2011). A genome assembly of *L.* kluyveri CBS 3082 was retrieved from GRYC^1^. Multiple sequence alignment of the consensus genotypes and genome assemblies was performed with the NASP pipeline (Sahl et al., 2016) using *L. fermentati* CBS 6772 as the reference genome. A matrix of single nucleotide polymorphisms (SNP) in the 7 strains was extracted from the aligned sequences. The SNPs were filtered so that only sites that were present in all 7 strains and with a minor allele frequency greater than 15% (one strain) were retained. The filtered matrix contained 11,517 SNPs at 6,330 sites. A maximum likelihood phylogenetic tree was estimated using IQ-TREE (Nguyen et al., 2015). IQ-TREE was run using the “GTR + ASC” model and 1000 ultrafast bootstrap replicates (Minh et al., 2013). The resulting maximum likelihood tree was visualized in FigTree and rooted with *L. kluyveri* CBS 3082. Haplotype phasing was attempted using WhatsHap (Supplementary Figure S1) (Martin et al., 2016), and by dividing haplotypes based on similarity to the reference genome as described by Ortiz-Merino et al. (2018).

### Strain Characterization

#### API Sugar Utilization Test

Substrate utilization test API ID 32C (BioMérieux, Marcy-l’Étoile, France) was used to analyze the biochemical spectrum of all *Lachancea fermentati* strains. Preparation of inoculum and inoculation of the strips was performed according to the manufacturers’ instructions. Colonies for the inoculum were grown on YPD agar plates for 48 h at 27°C. After inoculation, API ID 32C strips were incubated for 2 days at 28°C. The samples were evaluated visually by turbidity of the wells, differentiating positive (+), negative (-), and weak (w) growth.

#### Scanning Electron Microscopy

Yeast cultures for scanning electron microscopy (SEM) were prepared following the protocol for cultured microorganisms by Das Murtey and Ramasamy (2018). Single colonies were taken from YPD agar plate and grown in YPD broth for 24 h at 25°C. One milliliter of sample was centrifuged at 900 *g* for 2 min for pellet formation and resuspended in 5% glutaraldehyde solution prepared in 0.1 M phosphate buffer (pH 7.2) for fixation. After 30 min, the sample was centrifuged, the supernatant was discarded, and the pellet was washed twice in 0.1 M phosphate buffer. Consequently, the pellet was resuspended in 1% osmium tetroxide prepared in 0.1 M phosphate buffer. After 1 h, cells were again washed twice in 0.1 M phosphate buffer. The sample was then dehydrated through ethanol series of 35, 50, 75, 95%, absolute ethanol, and hexamethyldisilazane (HDMS) for 30 min per step (last two ethanol steps twice), centrifuging and discarding the supernatant for each change. Lastly, the second HDMS was discarded and the sample left drying overnight in a desiccator.

The dehydrated yeast sample was mounted onto plain aluminum stubs using carbon double surface adhesive and coated with a 5 nm gold-palladium (80:20) layer using a Gold Sputter Coater (BIO-RAD Polaron Division, SEM coating system, United Kingdom) and observed under a constant accelerating voltage of 5 kV under a JEOL scanning electron microscope type 5510 (JEOL, Tokyo, Japan).

#### Antifungal Susceptibility Test

Antifungal susceptibility was investigated using an agar-based method where a strip of inert material impregnated with a predefined concentration gradient of a single antifungal agent is used to directly quantify antifungal susceptibility in terms of an MIC (minimal inhibitory concentration) value, which corresponds to the growth inhibition in an elliptical zone. Antifungals tested were Amphotericin B, Fluconazole, Itraconazole, Voriconazole, Caspofungin, and Flucytosine, covering a wide range of antifungal mechanisms of action. Strips and RPMI agar plates were sourced from Liofilchem (Roseto degli Abruzzi, Italy). Yeast cultures were grown on Sabouraud dextrose agar for 48 h at 27°C. Well-isolated colonies were homogenized in sterile saline solution (0.85% NaCl) to obtain a turbidity equivalent to 0.5 McFarland standard. A sterile swab was soaked in the inoculum and used to streak the entire agar surface three times, rotating the plate 60° each time to ensure even distribution of the inoculum. The soaking and streaking procedure was repeated a second time. Strips were carefully applied on dry agar surface and plates were incubated at 35°C. Plates were read after 24, 48, and 72 h following the Etest antifungal reading guide (BioMérieux Antifungal Susceptibility Testing, 2019). The test was carried out in duplicate. If MICs differed between the duplicates, the higher MIC was reported.

#### POF Test

The phenolic off-flavor test was performed according to Meier-Dörnberg et al. (2017). Yeast strains were spread on yeast and mold agar plates (YM-agar) containing only one of the following precursors: either ferulic acid, cinnamic acid or coumaric acid. After 3 days of incubation at 25°C, plates were evaluated by a trained panel by sniffing to detect any of the following aromas: clove-like (4-vinylguajacol), Styrofoam-like (4-vinylstyrene) and medicinal-like (4-vinylphenol). *Saccharomyces cerevisiae* LeoBavaricus – TUM 68 (Research Center Weihenstephan for Brewing and Food Quality, Freising-Weihenstephan, Germany) was used as a positive control.

#### Flocculation Test

The flocculation test was performed using a slightly modified Helm’s assay (Bendiak et al., 1996; D’Hautcourt and Smart, 1999). Essentially, all cells were washed in EDTA and the sedimentation period was extended to 10 min. Wort was composed of 100 g/L spray-dried malt extract (Spraymalt Light, Muntons plc, Suffolk, United Kingdom) adjusted to 15 IBU (15 mg/mL iso-α-acids; from 30% stock solution; Barth-Haas Group, Nürnberg, Germany).

#### Stress Tests

Stress tests were performed on microplates through the repeated measurement of absorbance over a time period of 96 h (Multiskan FC, Thermo Fisher Scientific, Waltham, MA, United States).

The substrate for the hop sensitivity test was 75 g/L sterile-filtered wort adjusted to 0, 50, and 100 mg/L iso-α-acids (1 mg/L = 1 International Bitterness Unit, IBU), respectively by using an aliquot of a stock solution of 3% iso-α-acids in 96% (v/v) ethanol (Barth-Haas Group, Nürnberg, Germany).

For testing ethanol sensitivity, the sterile-filtered wort extract was adjusted to 0, 2.5, 5, 7.5, and 10% ABV with an aliquot of 100% (v/v) ethanol.

For testing pH sensitivity by lactic acid, the sterile-filtered wort was adjusted to pH ranges from 5.5 (no addition of acid) to 3.0 in steps of 0.5 with aliquots of 80% lactic acid (corresponding to lactic acid concentrations of 0; 1.7; 3.1; 6.1; 16.3; 48.4 mM).

For testing pH sensitivity by HCl, the sterile-filtered wort was adjusted to pH ranges from 5.5 (no addition of acid) to 1.5 in steps of 0.5 with aliquots of 2 M HCl.

Osmotic stress was tested by adjusting the sterile-filtered wort extract (100 g/L Muntons Spraymalt Light) to sorbitol concentrations of 0, 50, 100, 150, and 200 g/L, respectively.

For inoculation, strains were grown in sterilized wort for 24 h at 25°C under aerobic conditions. The microtiter plate wells were inoculated with a concentration of 10^5^ cells/mL. The wells contained 200 μL of the respective wort substrates. Plates were incubated at 25°C and absorbance was measured every 30 min at 600 nm without shaking over a time period of 96 h (Multiskan FC, Thermo Fisher Scientific, Waltham, MA, United States).

### Fermentation Trial

Single colonies of the respective strains were taken from YPD agar plates after 72 h growth at 25°C and transferred into a 250 mL sterile Duran glass bottle (Lennox Laboratory Supplies Ltd., Dublin, Ireland) containing 150 mL propagation wort consisting of 75 g/L spray-dried malt and 30 g/L glucose (Gem Pack Foods Ltd., Dublin, Ireland), sterilized at 121°C for 15 min. The bottles were covered with sterile cotton and placed in an incubator with orbital shaker (ES-80 shaker-incubator, Grant Instruments (Cambridge) Ltd., Shepreth, United Kingdom) and incubated for 48 h at an orbital agitation of 170 rpm at 25°C. Cell count was performed using a Thoma Hemocytometer with a depth of 0.1 mm (Blaubrand, Sigma-Aldrich, St. Louis, MO, United States).

Fermentation wort was prepared by dissolving 100 g/L spray-dried malt extract in 1 L of brewing water and sterilized at 121°C for 15 min followed by filtration through sterile grade 1V Whatman filter (Whatman plc, Maidstone, United Kingdom) to remove hot trub built up during sterilization. Iso-α-acids were added to the wort at a concentration of 15 mg/L (15 IBU).

Fermentation trials were carried out in 250 mL sterile Duran glass bottles, equipped with an air lock. Bottles were filled with 150 mL of wort. Yeast cells for pitching were washed by centrifugation at 900 *g* for 5 min and resuspended in sterile water to ensure no carryover of sugars or acids from the propagation wort into the fermentation wort. Pitching rate was 10^7^ cells/mL. Fermentation temperature was 25°C. Fermentation was performed until no change in extract could be measured for two consecutive days.

### Lactic Acid Production Optimization of KBI 12.1

#### Response Surface Methodology (RSM)

To investigate lactic acid production performance by KBI 12.1 at different fermentation parameters, RSM was performed using DesignExpert 9 software (StatEase, Minneapolis, MN, United States). A three factorial, face-centered, central composite design with duplicate factorial points and 6 replications of the center point was chosen. The predictor factors were extract (5, 10, 15°P), temperature (16, 22, 28°C), and pitching rate (5, 32.5, 60 × 10^6^ cells/mL).

Spray-dried malt extract served as substrate. Wort preparation, propagation and inoculation was carried out as outlined in 2.4. Sterilized and filtered wort extract of 15°P was used as the base and diluted with sterile water when necessary. Fermentation volume was 150 mL in 250 mL Duran glass bottles equipped with an air lock. Fermentation was performed until no change in extract could be measured for two consecutive days.

#### Spiked Glucose Trial

Wort preparation, propagation and inoculation was carried out as outlined in 2.4. The 7°P wort was produced from 75 g wort extract in 1 L of water. The 7°P wort plus 3% glucose was produced from 75 g wort extract and 30 g glucose in 1 L of water. The 10°P wort was produced by dilution of the 15°P wort from 2.5.1 with water. Fermentation volume was 150 mL in 250 mL Duran glass bottles equipped with an air lock. Pitching rate was 5 × 10^6^ cells/mL and fermentation temperature was 25°C. Fermentation was performed until no change in extract could be measured for two consecutive days.

### Pilot Brew

Wort for the pilot brew was produced in a 60 L pilot-scale brewing plant consisting of a combined mash-boiling vessel, a lauter tun and whirlpool (FOODING Nahrungsmitteltechnik GmbH, Stuttgart, Germany). Weyermann Pilsner Malt was milled with a two-roller mill (“Derby,” Engl Maschinen, Schwebheim, Germany) at a 0.8 mm gap size. Seven kilograms of crushed malt was mashed in with 30 L of brewing water at 50°C. To increase the amount of glucose, 7 g of Amylo 300 (Kerry Group, Tralee, Ireland) were added at the begin of mashing (1 g/kg of malt). The following mashing regime was employed: 20 min at 50°C, 60 min at 65°C and 5 min at 78°C. The mash was pumped into the lauter tun and lautering was performed after a 15 min lauter rest, employing four sparging steps of 5 L hot brewing water each. Boil volume was 50 L at a gravity of 1.038 (9.9°P). At the start of the boil, 15 g of Magnum hop pellets (10.5% iso-α-acids) were added for a calculated IBU content of 6.5. Total boiling time was 45 min. After boiling, gravity was adjusted to 1.034 (8.5°P) with hot brewing water, and hot trub precipitates and hop residue were removed in the whirlpool with a rest of 20 min. Clear wort was pumped through a heat exchanger and filled into 60 L fermentation vessels at a temperature of 25°C.

Yeast was pitched at a pitching rate of 5 × 10^6^ cells/mL. Fermentation temperature was 25 ± 1°C (uncontrolled). Samples were taken every 12 h. After 36 h, 30 L of the young beer were filtered through a plate filter (Seitz K 200; Pall GmbH, Dreieich, Germany) to stop fermentation by removing the yeast, and filled into a 50 L keg. The remaining young beer was left in the fermenter to reach final attenuation. To carbonate the kegged beer, the keg was repeatedly topped up with CO_2_ at a pressure of 1 bar at 2°C. Ten days after stopping fermentation, the carbonated beer was filled into 330 mL brown glass bottles with a counter-pressure hand-filler (TOPINCN, Shenzen, China) and capped. Bottles were pasteurized in a pilot retort (APR-95; Surdry, Abadiano, Vizcaya, Spain) with spray water at 65°C for 10 min resulting in approximately 23 pasteurization units (PU). Beer bottles were stored in a dark place at 2°C for further analysis and sensory evaluation.

### Sensory

The low alcohol *Lachancea* beer produced at pilot scale (bottled beer) was tasted and judged by a sensory panel of 15 experienced panelists. The panelists were asked to evaluate the intensity of fruitiness in aroma, the sweetness/acidity ratio (0 “too sweet”; 5 “just right”; 10 “too sour”) and the general acceptability of the low alcohol beer on a scale from 0, “not acceptable,” to 10, “extremely acceptable.” Samples were served at a temperature of 12°C.

### Analytics

#### HPLC Analyses

The cell-free supernatant of fermented samples was analyzed using the following methods. Sugars and ethanol were determined by high performance liquid chromatography (HPLC) Agilent 1260 Infinity (Agilent Technologies, Santa Clara, CA, United States) equipped a refractive index detector (RID) and a Sugar-Pak I 10 μm, 6.5 mm × 300 mm column (Waters, Milford, MA, United States) with 50 mg/L Ca-EDTA as mobile phase and a flow rate of 0.5 mL/min at 80°C. Differentiation of maltose and sucrose was achieved with a Nova-Pak 4 μm, 4.6 mm × 250 mm column (Waters, Milford, MA, United States) with acetonitrile/water 78:22 (v/v) as mobile phase and a flow rate of 1.0 mL/min. Lactic acid was quantified via HPLC (DIONEX UltiMate 3000, Thermo Fisher Scientific, Waltham, MA, United States) with diode array detector (DAD) and a Hi-Plex H 8 μm, 7.7 mm × 300 mm column (Agilent Technologies, Santa Clara, CA, United States) with 5 mM H_2_SO_4_ as mobile phase and a flow rate of 0.5 mL/min at 60°C. Quantification was achieved by external standards in a calibration range of 0.5–30 mM.

#### Volatiles Analysis by GC-MS

Analysis of volatiles in the cell-free supernatant of the fermented samples was carried out as follows. Analytes were extracted using liquid-liquid extraction with Methyl-tert-butyl ether directly in the vial. Analysis was performed using a mid-polarity column (Zebron ZB-1701, GC Cap. Column 30 m × 0.25 mm × 0.25 μm; Phenomenex, Torrance, CA, United States) installed in a GC 7890B (Agilent Technologies, Santa Clara, CA, United States) coupled with a quadrupole detector 5977B (Agilent Technologies, Santa Clara, CA, United States). The system was controlled by ChemStation (Agilent Technologies, Santa Clara, CA, United States). The GC-method was set up as described by Pinu and Villas-Boas (2017) with only minor modifications. Samples were analyzed in Selected Ion Monitoring (SIM) mode. Quantifications were performed using external calibration lines.

#### Free Amino Nitrogen

Free amino nitrogen (FAN) was measured using a ninhydrin-based dying method, where absorbance is measured at 570 nm against a glycine standard (ASBC Method Wort-12 A).

#### Statistical Analysis

Fermentations and analyses were carried out in triplicate, unless stated otherwise. Statistical analysis was performed using RStudio, Version 1.1.463 with R version 3.5.2 (RStudio Inc., Boston, MA, United States; R Core Team, r-project). One-way ANOVA was used to compare means and Tukey’s test with 95% confidence intervals was applied for the pairwise comparison of means. Principal component analysis (PCA) was performed with the R packages FactoMineR and Factoshiny (Le et al., 2008). Values are given as the mean ± standard deviation.

## Results

The *Lachancea fermentati* strains investigated in this study were isolated from four individual kombucha cultures. KBI 1.2 and KBI 3.2 originate from the Conterminous United States, while KBI 12.1 originates from Hawaii, and KBI 5.3 originates from a kombucha culture from Australia. They were identified as *Lachancea fermentati* strains via sequencing and comparing the D1/D2 region of the large subunit rDNA to the public NCBI nucleotide database^2^ (Bellut et al., 2019a). The country of origin of the strain CBS 707 is unknown. CBS 6772 was isolated from a spoiled strawberry beverage in South Korea (Table 1).

### Genomics

**FIGURE 1:**
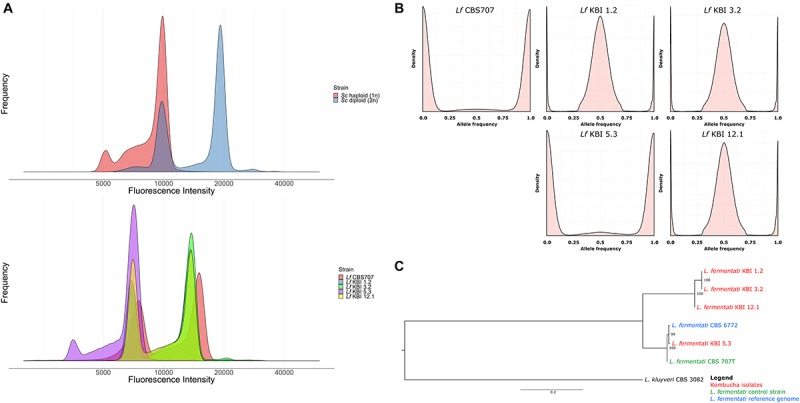
**(A)** Fluorescence intensity of SYTOX Green-stained haploid and diploid *S. cerevisiae* control strains and the five *L. fermentati* strains during flow cytometry. **(B)** The allele frequency distributions of the single nucleotide variants observed in the five sequenced *L. fermentati* strains. Peaks only at 0 and 1 suggest a single allele at each site, while peaks at 0, 0.5, and 1 suggest two alleles at each site. **(C)** A maximum likelihood phylogenetic tree based on SNPs at 6330 sites in the six *L. fermentati* and one *L. kluyveri* genomes (rooted with *L. kluyveri* as outgroup). Numbers at nodes indicate bootstrap support values. Branch lengths represent the number of substitutions per site.

To better understand the variation in these four strains, whole-genome sequencing of the four *L. fermentati* kombucha isolates and the CBS707 type strain was performed. These were sequenced with 150 bp paired-end Illumina sequencing to an average coverage ranging from 115× to 139×. Reads were aligned to the reference genome of *L. fermentati* CBS 6772, and single nucleotide polymorphisms (SNPs) were called with FreeBayes. A total of 370,027 variable sites were observed across the five strains compared to the reference genome. Interestingly, a high number of SNPs (>250,000) were observed in the three kombucha isolates originating from the United States (Table 1). This corresponds to a nucleotide sequence divergence around 2.4–2.7% in the 10.3 Mbp genome. The majority of these SNPs were heterozygous (>2% heterozygosity), suggesting that not only were these strains non-haploid, but possessed divergent genotypes. Allele frequency peaks at 0, 0.5, and 1, suggested that these strains were diploid (Figure 1B). The heterozygosity was considerably higher than what was observed, for example, in any of the recently sequenced 1,011 *S. cerevisiae* strains (Peter et al., 2018) or 14 *Kluyveromyces marxianus* strains (Ortiz-Merino et al., 2018). The kombucha isolate originating from Australia, KBI 5.3, and the CBS707 type strain had around 20,000 SNPs compared to the reference genome. Here, the majority of the SNPs were homozygous, suggesting that the strains were either haploid or homozygous diploids (Figure 1B). The average pairwise nucleotide diversity (π) in this limited set of strains was 0.0126, which is comparable to what has been observed for *Kluyveromyces marxianus* (Ortiz-Merino et al., 2018) and slightly higher than for a wild population of *Lachancea quebecensis*. The three heterozygous kombucha isolates also contained several regions where heterozygosity was lost (Supplementary Figure S1). Common regions, where loss of heterozygosity (LOH) was observed in all three strains, could be found across chromosome G and on the right arm of chromosome F. In addition, KBI 12.1 had large LOH regions on the left arms of both chromosome F and H.

Flow cytometry was used to confirm the ploidy of the strains. The natural ploidy of *L. fermentati* and other members of the *Lachancea* genus appears to vary, with reports of both haploid and diploid strains (Souciet et al., 2009; Agier et al., 2013; Freel et al., 2014; Banilas et al., 2016; Hranilovic et al., 2017). Here, the three heterozygous kombucha isolates appeared diploid, while KBI 5.3 appeared haploid (Figure 1A). Despite the lack of heterozygous SNPs, the CBS707 type strain also appeared diploid. This is in line with what has previously been reported for the strain (Agier et al., 2013). Read coverage also suggested that CBS 707 also harbored an extra third copy of chromosome C, while no aneuploidy was observed in any of the kombucha isolates (Supplementary Figure S2). Fluorescence intensities of the *L. fermentati* strains during flow cytometry were slightly lower than those of haploid and diploid *S. cerevisiae* references, as can be expected based on the smaller genome size of *L. fermentati.*

Phylogenetic analysis based on the single nucleotide variants that were observed in the four kombucha isolates and the CBS 707 type strain, separated the three heterozygous kombucha isolates (KBI 1.2, KBI 3.2, and KBI 12.1) into a separate clade from the one containing CBS 707, CBS 6772, and KBI 5.3 (Figure 1C). Because of the high heterozygosity, which can skew the results, we attempted to separate the two haplotypes both using variant phasing with WhatsHap and by assigning the haplotypes based on similarity to the reference genome as described by Ortiz-Merino et al. (2018). In both cases, one haplotype could be found together with CBS 707, CBS 6772, and KBI 5.3, while the other haplotype formed a separate clade (Supplementary Figure S3). It is therefore likely that the heterozygous kombucha isolates have emerged through breeding between strains from two different *L. fermentati* populations. To ensure that the heterozygous strains were not interspecific hybrids, sequencing reads were also aligned to a concatenated reference genome consisting of the genomes of 12 species in the *Lachancea* genus. Reads aligned almost exclusively to the *L. fermentati* genome, confirming that they were not interspecific hybrids (Supplementary Figure S4).

### Yeast Characterization

#### API Sugar Utilization, Flocculation, and POF Test

The API sugar utilization test was performed to investigate intraspecific differences. The results are shown in Table 2, alongside the results from the flocculation test and phenolic off-flavor test.

**TABLE 2:** API sugar utilization test.

**Substrate/Assay**	**CBS 707**	**KBI 1.2**	**KBI 3.2**	**KBI 5.3**	**KBI 12.1**
Control	−	−	−	−	−
D-Galactose	+	+	+	+	+
Cycloheximide (Actidione)	+	+	+	+	+
D-Saccharose	+	+	+	+	+
Lactic acid	−^1^	w	w	−	w
D-Cellobiose	+	w	w	−	+
D-Raffinose	+	+	+	+	+
D-Maltose	+	+	+	+	+
D-Trehalose	+	+	+	+	+
Potassium 2-Ketogluconate	w	w	w	w	w
Methyl-αD-Glucopyranoside	+	+	+	+	+
D-Mannitol	+	+	+	w	+
D-Sorbitol	+	+	+	+	+
Palatinose	+	+	+	+	+
D-Melezitose	w	+	w	w	+
Potassium gluconate	w	−	w	−	w
D-Glucose	+	+	+	+	+
L-Sorbose	w	+	w	+	+
Esculin ferric citrate	w	+	+	w	+
Flocculation (%)	15 ± 2^a^	88 ± 10^b^	28 ± 1^a^	25 ± 8^a^	20 ± 6^a^
Definition	Non-flocculent	Very flocculent	Moderately flocculent	Moderately flocculent	Moderately flocculent
Phenolic off-flavor	Negative	Negative	Negative	Negative	Negative

*− negative; + positive; w weak. Substrates negative for all strains are not included in the table. Different letters in superscripts indicate a significant difference of the means within a row (p ≤ 0.05). The full table of substrates is included in Supplementary Data Sheet S1. ^1^Deviation from literature which states a positive reaction (Kurtzman et al., 2011).*

The sugar utilization pattern showed minor differences. The type strain CBS 707 and KBI 5.3 showed no growth with lactic acid as substrate, whereas KBI 1.2, KBI 3.2, and KBI 12.1, exhibited weak growth. However, Kurtzman et al. (2011) reported positive growth for CBS 707. Esculin ferric citrate was positive for KBI 1.2, KBI 3.2, and KBI 12.1 and weak for CBS 707 and KBI 5.3. The color reaction resulting from a positive reaction to esculin ferric citrate is associated with β-glucosidase activity (Pérez et al., 2011). However, cellobiose, a β-1,4-linked sugar, was not metabolized by KBI 5.3 and only weakly by KBI 1.2 and KBI 5.3 despite showing weak or positive reactions to esculin ferric citrate. In a study on *Lachancea fermentati* wine strains, Porter (2017) reported that from 10 tested strains, 80% showed β-glucosidase activity.

According to the modified Helm’s assay, CBS 707, KBI 3.2, KBI 5.3, and KBI 12.1 showed low flocculation between 15 and 28%, with no statistically significant difference (*p* ≤ 0.05). KBI 1.2 showed, with 88%, the highest flocculation behavior. Flocculation of *Lachancea fermentati* strains has also been reported in other studies and its degree was shown to be strain-dependent (Romano and Suzzi, 1993; Porter, 2017; Porter et al., 2019a). However, yeast flocculation assays like the Helm’s assay can deviate from observations on flocculation behavior in practice and can be difficult to reproduce (Stewart, 2018). In a previous study by Bellut et al. (2019a), KBI 12.1 exhibited high flocculation >80%. In fact, from observations during fermentation trials in this study, KBI 12.1 shows a more flocculent behavior than the results of the Helm’s assay suggest here, with flocculation more comparable to that of the brewers’ yeast WLP001 as previously reported (Bellut et al., 2019a). All strains showed negative POF behavior.

#### Scanning Electron Microscopy (SEM)

**FIGURE 2:**
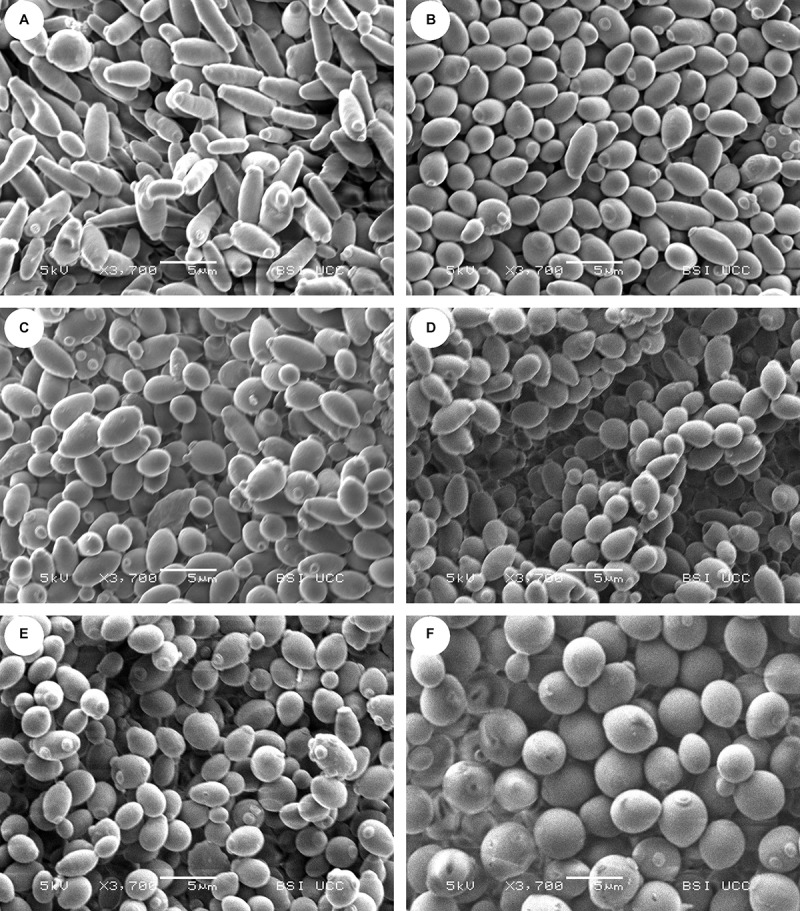
Scanning electron microscopy (SEM) pictures of the yeast strains **(A)** CBS 707, **(B)** KBI 1.2, **(C)** KBI 3.2, **(D)** KBI 5.3, **(E)** KBI 12.1, and **(F)** WLP001 at same magnification of ×3,700. Size of horizontal bar: 5 μm.

To visualize the different yeast strains and to investigate differences in cell morphology, scanning electron microscopy (SEM) was performed. The SEM pictures of the strains can be seen in Figure 2.

The SEM confirmed inter- and intraspecific differences in cell morphology that had been suspected from observations under the light microscope. The almost rod-shaped cells of the type strain CBS 707 were longer and thinner than the other *Lachancea fermentati* KBI strains. Bud scars appeared to be mostly located at or near the ends of the rod-shape. The KBI strains seemed to have a rounder shape compared to the type strain. KBI 12.1 appeared to exhibit the highest proportion of oval or spherical shaped cells of the *Lachancea fermentati* strains, while cells of WLP001 showed a substantially more pronounced spherical shape. Regarding cell size, the cells of the brewers’ yeast were larger compared to the *Lachancea fermentati* cells. The cell size is related to the total surface area of the cell, which determines import and export rates of nutrients and fermentation products (Michel et al., 2017). The difference in cell size can therefore have a strong effect on fermentation performance and must be considered when choosing pitching rates.

#### Stress Tests

During fermentation, yeast strains applied in brewing must deal with several stress factors. Iso-α-acid concentrations of 100 and more mg/L are no longer a rarity [e.g., strong India Pale Ales (IPAs)]. Ethanol, another stressor, accumulates during fermentation, especially in high gravity brewing, which by itself involves another stress factor: osmotic stress (here simulated with sorbitol). Sour beers are also gaining popularity and yeasts are required to ferment wort with a low pH and high initial lactic acid concentration (Peyer et al., 2017). Additionally, strains of the *Lachancea* genus can possess the ability to produce significant amounts of lactic acid during alcoholic fermentation. The stress tests were performed to investigate inter- and intraspecific differences. Table 3 shows the results of the relative growth in wort in microtiter analyses at a snapshot at 48 h after pitching with, and without the stressor in different concentrations.

**TABLE 3:** Relative growth in percent in wort after 48 h with and without stressor in different concentrations based on OD_600_ measurements.

**Stress factor (Unit)**	**Concentration**	**CBS 707**	**KBI 1.2**	**KBI 3.2**	**KBI 5.3**	**KBI 12.1**	**WLP001**
Hops	0	100	100	100	100	100	100
(IBU)	50	**107**	103	99	100	105	**95**
	100	105	99	99	101	105	98
Ethanol	0	100	100	100	100	100	100
(% ABV)	2.5	**90**	98	97	95	96	95
	5	**77**	87	**87**	**86**	**85**	**76**
	7.5	**5**	**53**	**70**	**66**	**71**	**16**
	10	**0**	**0**	**25**	**8**	**4**	**0**
Sorbitol	0	100	100	100	100	100	100
(g/L)	50	89	89	90	92	**86**	99
	100	**64**	**77**	**78**	**78**	**72**	91
	150	**35**	**52**	**54**	**62**	**46**	83
	200	26	**35**	**32**	**41**	35	**70**
Lactic	5.5	100	100	100	100	100	100
acid (pH)	5	**96**	99	99	99	97	100
	4.5	95	**97**	**97**	**98**	98	101
	4	96	98	98	98	98	104
	3.5	95	97	95	**95**	98	100
	3	**84**	**87**	**83**	**85**	**86**	**53**
HCl	5.5	100	100	100	100	100	100
(pH)	5	101	100	102	101	99	**105**
	4.5	102	101	100	102	100	104
	4	101	102	100	102	98	108
	3.5	98	**98**	**97**	**98**	97	105
	3	**93**	**88**	**87**	**91**	**89**	**85**
	2.5	**71**	**71**	**69**	**79**	**72**	**0**
	2	**1**	**5**	**1**	**1**	**2**	1
	1.5	1	**1**	1	1	1	1

*Bold values are significantly different from the previous value within a stress test for the individual strain (p ≤ 0.05).*

The concentration of iso-α-acids did not have an influence of the growth of the strains which is in accordance with previous reports on various non-*Saccharomyces* species (Bellut et al., 2018). However, Michel et al. (2016) reported the presence of 90 IBU to affect *Torulaspora delbrueckii* strains, resulting in a slightly prolonged lag phase and slightly decreased slope of the growth curve.

Among the *L. fermentati* strains, the KBI strains exhibited a greater tolerance toward higher ethanol concentrations in the wort compared to CBS 707, which showed a small but significant growth impairment already at 2.5% ABV, manifesting as a 10% decreased relative growth. At 7.5% ABV, CBS 707 showed almost full growth inhibition (5% relative growth remaining) while the KBI strains still showed relative growth between 53 and 71%. At an ABV of 10% in the wort, growth of CBS 707 and KBI 1.2 was fully inhibited, while KBI 3.2, KBI 5.3, and KBI 12.1 still exhibited little growth, at 4–25%, with KBI 3.2 being the most ethanol tolerant strain. In accordance, Porter et al. (2019b) observed full inhibition of a *L. fermentati* strain at 10% ABV during a growth test on agar while it still exhibited growth at 7% ABV. The brewers’ yeast WLP001 showed significant inhibition at 5% ABV with 24% decreased relative growth. Full growth inhibition was reached at 10% ABV. Overall, WLP001 showed a greater sensitivity toward ethanol compared to the KBI strains.

During osmotic stress, at the presence of high concentrations of sorbitol, the *Lachancea fermentati* strains showed a greater growth impairment compared to WLP001 with only 26–41% remaining relative growth at 200 g/L sorbitol compared to 70% for WLP001. Intraspecific differences in growth inhibition among the *Lachancea fermentati* were generally low, CBS 707 tentatively showing greater sensitivity.

In the presence of lactic acid, all yeast strains were resilient against concentrations of up to 16.3 mM (pH 3.5). Although statistically significant, growth impairment at lactic acid concentrations between 1.7 and 16.3 mM showed to be very low with a maximum decrease in relative growth by 5%. Only at extreme lactic acid concentrations of 48.4 mM (pH 3), did the *L. fermentati* strains show slight growth impairment of 13–17%, while WLP001 exhibited a growth impairment of 47%.

When the wort pH was adjusted with HCl, the strains showed less sensitivity compared to the pH adjustment with lactic acid. For example, at pH 3, WLP001 still exhibited 85% growth compared to 53% at pH 3 when adjusted with lactic acid. However, at pH 2.5 and lower, WLP001 growth was fully inhibited while the *L. fermentati* strains still exhibited relative growth between 72 and 79%. Full growth inhibition of the *L. fermentati* strains was reached at pH 2. Intraspecific differences among the *Lachancea fermentati* strains were small.

Differences in growth impairment by the different acids at same pH can be explained with the chemical property of weak acids. The presence of a weak acid like lactic acid leads to an increased stress for the yeast cell. The lower the extracellular pH, the more lactic acid is present in its protonated form, especially at a pH below the pK_a_ of the respective acid (lactic acid pK_a_: 3.86) and can therefore enter the cell via passive diffusion. Inside the cell, at a higher intercellular pH, lactic acid is deprotonated. Consequently, the cell must export the proton as well as the anion, creating an energy-requiring cycle. At high concentrations, this mechanism can lead to the dissipation of the proton motive force, leading to cell death (Colombié et al., 2003; Sauer et al., 2010).

#### Antifungal Susceptibility

While *Candida* species are the lead cause for fungemia, cases of non-pathogenic species such as *Saccharomyces cerevisiae* acting as opportunistic pathogens in immunocompromised hosts have been reported (Cassone et al., 2003; Hamoud et al., 2011) and one case of fungemia caused by *Lachancea fermentati* in an immunocompromised host has also been recorded (Leuck et al., 2014). Also, given the fact that *Lachancea* species are capable of growth at human body temperature (37°C) (Kurtzman et al., 2011), it is reasonable to investigate potential resistances against antifungal agents. The minimal inhibitory concentration (MIC) of a range of antifungal agents was tested by Etest. The results are shown in Table 4. All strains showed to be susceptible to all classes of antifungal agents with only small intra- and interspecific differences.

**TABLE 4:** Minimal inhibitory concentration (MIC) of selected antifungal agents after 24 h of incubation.

**Antifungal agent**	**Range**	**CBS 707**	**KBI 1.2**	**KBI 3.2**	**KBI 5.3**	**KBI 12.1**	**WLP001**
Amphotericin B	0.002–32	0.032	0.094	0.094	0.125	0.094	1
Caspofungin	0.002–32	1	1.5	1.5	1.5	1.5	0.5
Flucytosine	0.002–32	0.094	0.064	0.125	0.125	0.064	0.023
Fluconazole	0.016–256	12	12	12	12	12	24
Itraconazole	0.002–32	0.5	0.75	1	1	0.75	1
Voriconazole	0.002–32	0.094	0.125	0.125	0.125	0.125	0.125

*Values in μg/mL.*

### Fermentation Trials

#### Fermentation of Wort

Fermentation trials were conducted to investigate strain performances in terms of ethanol and lactic acid production and the concentration of fermentation by-products. Spray-dried wort extract from barley malt served as the substrate for all fermentations. Table 5 shows the analytical parameters of the fermentation wort including extract, pH, free amino nitrogen (FAN) and sugar concentration.

**TABLE 5:** Analysis of fermentation wort.

Extract	°P	9.40 ± 0.00
pH		4.99 ± 0.01
FAN	mg/L	99 ± 1
Fructose	g/L	1.78 ± 0.02
Glucose	g/L	8.53 ± 0.05
Sucrose	g/L	1.02 ± 0.01
Maltose	g/L	40.64 ± 0.25
Maltotriose	g/L	11.94 ± 0.07

##### Analysis of fermented samples

Fermentation was carried out until no change in extract was measurable for two consecutive days. For CBS 707, KBI 1.2, KBI 5.3, and WLP001, final attenuation was reached after 11 days of fermentation at 25°C. KBI 3.2 and KBI 12.1 reached final attenuation after 13 days of fermentation. Table 6 shows the analytical results of the fermentation trials.

**TABLE 6:** Analysis of fermented worts.

	**Attribute**	**Unit**	**CBS 707**	**KBI 1.2**	**KBI 3.2**	**KBI 5.3**	**KBI 12.1**	**WLP001**
	Attenuation		70%0%^b^	70%0%^b^	68%1%^b^	70%0%^b^	55%2%^a^	85%1%^c^
	App. extract	°P	2.830.03^b^	2.830.03^b^	2.990.14^b^	2.790.01^b^	4.270.22^c^	1.380.06^a^
	Real extract	°P	4.140.01^b^	4.170.02^b^	4.270.11^b^	4.120.01^b^	5.310.18^c^	2.940.08^a^
	Ethanol	% ABV	3.730.01^b^	3.730.04^b^	3.630.12^b^	3.760.03^b^	2.960.11^a^	4.420.02^c^
	pH		4.240.02^d^	4.270.01^de^	4.130.02^c^	4.310.01^e^	3.950.01^a^	4.070.02^b^
	Lactic acid	mM	2.410.02*e*	1.550.03*c*	1.820.03*d*	1.330.01*b*	3.470.12*f*	0.940.02*a*
	FAN	mg/L	824^bc^	822^bc^	804^bc^	733^b^	831^c^	524^a^
Sugar	Fructose		92%0%^a^	100%^b^	100%^b^	98%4%^b^	100%^b^	100%^b^
consumption	Glucose		100%	100%	100%	100%	100%	100%
	Sucrose		100%	100%	100%	100%	100%	100%
	Maltose		100%^b^	100%^b^	98%2%^b^	100%^b^	76%4%^a^	100%^b^
	Maltotriose		−4%1%^a^	−8%1%^a^	−5%4%^a^	−10%1%^a^	3%1%^b^	81%1%^c^
Fermentation	Diacetyl	mg/L	<LOD	<LOD	0.020.02^a^	<LOD	0.020.00^a^	<LOD
by-products	Ethyl acetate	mg/L	14.350.20^d^	13.720.28^d^	14.010.68^d^	9.060.21^b^	11.700.82^c^	7.060.50^a^
	3-Methylbutyl acetate	mg/L	0.480.06^a^	0.360.08^a^	0.400.03^a^	0.430.13^a^	0.290.05^a^	0.300.04^a^
	2-Phenylethyl acetate	mg/L	0.080.01^a^	0.520.03^c^	0.570.02^c^	0.130.02^a^	0.440.04^b^	0.080.02^a^
	Σ Esters	mg/L	16.140.31^c^	16.020.40^c^	16.100.64^c^	10.840.38^a^	13.610.99^b^	9.520.47^a^
	Σ Alcohols	mg/L	119.987.87^d^	77.831.64^ab^	86.354.81^bc^	81.424.72^bc^	65.612.34^a^	93.396.19^c^

*Sugars are given in percent consumption of the initial amount. 100% consumption indicates a concentration below the limit of detection (LOD). Values are shown as means ± standard deviation. Different letters in superscripts indicate a significant difference of the means within a row (p ≤ 0.05).*

The *L. fermentati* strains reached final attenuations of 70% and lower, owing to their inability to consume maltotriose. KBI 12.1 exhibited, at 55%, the lowest attenuation. Sugar analysis revealed that KBI 12.1 had only used up 76% of maltose while the other strains had depleted it by the end of fermentation. Only WLP001 consumed maltotriose, at 81%, while the *L. fermentati* strains did not consume any maltotriose. At the end of fermentation, slightly higher values for maltotriose than the initial values were detected in some of the worts fermented with the *L. fermentati* strains. Glucose and sucrose were completely consumed by all strains by the end of fermentation. In the wort fermented with CBS 707, a small amount of fructose remained.

Ethanol concentrations correlated with attenuation. The brewers’ yeast WLP001 exhibited the highest concentration, at 4.4% ABV, followed by four of the *L. fermentati* strains at around 3.7% ABV. KBI 12.1 produced only 3.0% ABV.

Lactic acid concentrations reached 0.94 mM in the sample fermented with WLP001. Li and Liu (2015) reported similar values produced by a lager yeast, at 1.03 mM. The *Lachancea* yeasts exhibited significantly higher final lactic acid values. KBI 12.1 exhibited the highest lactic acid concentration, at 3.47 mM, followed by CBS 707, KBI 3.2, KBI 1.2, and KBI 5.3, at 2.41, 1.82, 1.55, and 1.33 mM, respectively. However, these values were still below the reported flavor threshold of lactic acid in beer of 4.44 mM (400 mg/L) (Siebert, 1999).

FAN consumption by the *L- fermentati* strains was relatively low with 70 or 80% of the initial amount remaining by the end of fermentation. By comparison, WLP001 consumed half of the amount of FAN in the wort. The pattern of a low FAN consumption of non-*Saccharomyces* yeasts compared to brewers’ yeasts has been observed in previous studies (Estela-Escalante et al., 2016; Bellut et al., 2018). It has mostly been attributed to a less intensive fermentation due to limited sugar consumption, however, in this study, fermentation and sugar consumption did not differ to an extent that would account for the reduced FAN uptake, suggesting an alternative cause.

**FIGURE 3:**
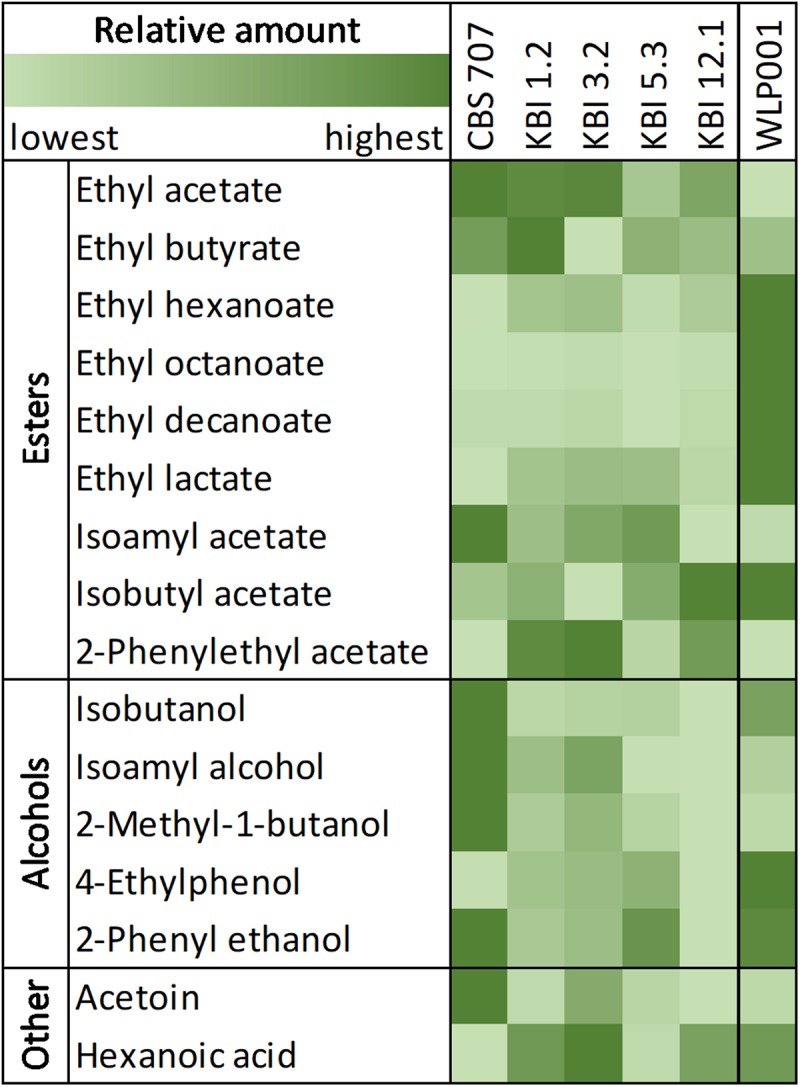
Heatmap of relative amount of volatile compounds in the fermented worts. A full table of relative and quantified compounds can be found in Supplementary Data Sheet S1.

When detected, diacetyl values were, at 0.02 mg/L, at or below the flavor threshold of 0.02–0.10 mg/L (Meilgaard, 1975; Saison et al., 2009). Ethyl acetate values were significantly higher in the *L. fermentati* strains compared to WLP001, up to double the concentration. 3-Methylbutyl acetate (isoamyl acetate) concentrations were similar among all strains. 2-Phenylethyl acetate concentrations were significantly higher in KBI 1.2, KBI 3.2, and KBI 12.1 compared to the other strains. CBS 707 produced the highest amount of higher alcohols, at 120 mg/L, and KBI 12.1 produced the lowest amount, at 66 mg/L. Figure 3 illustrates the relative amounts of volatile fermentation by-products produced by the different yeast strains. WLP001 produced higher amounts of the higher ethyl esters (i.e., ethyl hexanoate, ethyl octanoate, ethyl decanoate) while CBS 707 produced more higher alcohols compared to the other strains. Interestingly, despite the increased lactic acid production by the *Lachancea* strains, ethyl lactate concentrations were higher in the wort fermented with WLP001. None of the volatile fermentation by-products were detected in concentrations above their individual flavor thresholds (Supplementary Data Sheet S1).

#### Lactic Acid Production Optimization With KBI 12.1

While the *L. fermentati* strains produced significantly higher amounts of lactic acid compared to the *S. cerevisiae* control, the values were still below the reported flavor threshold for beer of 4.44 mM (400 mg/L) (Siebert, 1999). Therefore, we applied RSM and conducted a trial in wort extract with spiked glucose to enhance lactic acid production of strain KBI 12.1, which was chosen as the highest lactic acid producer from the screening (Table 6).

##### Response surface methodology.

Non-*Saccharomyces* yeasts can require a significantly higher pitching rate to show good fermentation performance compared to brewers’ yeast due to their typically smaller cell size. A study by Michel et al. (2017) using RSM to optimize fermentation conditions of a *Torulaspora delbrueckii* strain in wort showed that high sensorial desirability of the produced beer was achieved at a high pitching rate of 60 × 10^6^ cells/mL. Furthermore, the fermentation temperature can have significant influences on the production of fermentation by-products across yeast genera, e.g., a higher temperature resulting in increased ester production (Verstrepen et al., 2003; Michel et al., 2017; Tyrawa et al., 2019).

To investigate the influences of the fermentation parameters: pitching rate, temperature and starting extract, on the production of lactic acid, RSM was applied. A three factorial, face-centered, central composite design was chosen to investigate the lactic acid production by KBI 12.1 in wort extract in the range of extract content between 5 and 15°P, a pitching rate between 5 and 60 × 10^6^ cells/mL, and a fermentation temperature between 16 and 28°C. The detailed experiment design and model statistics are shown in Supplementary Data Sheet S2.

**FIGURE 4:**
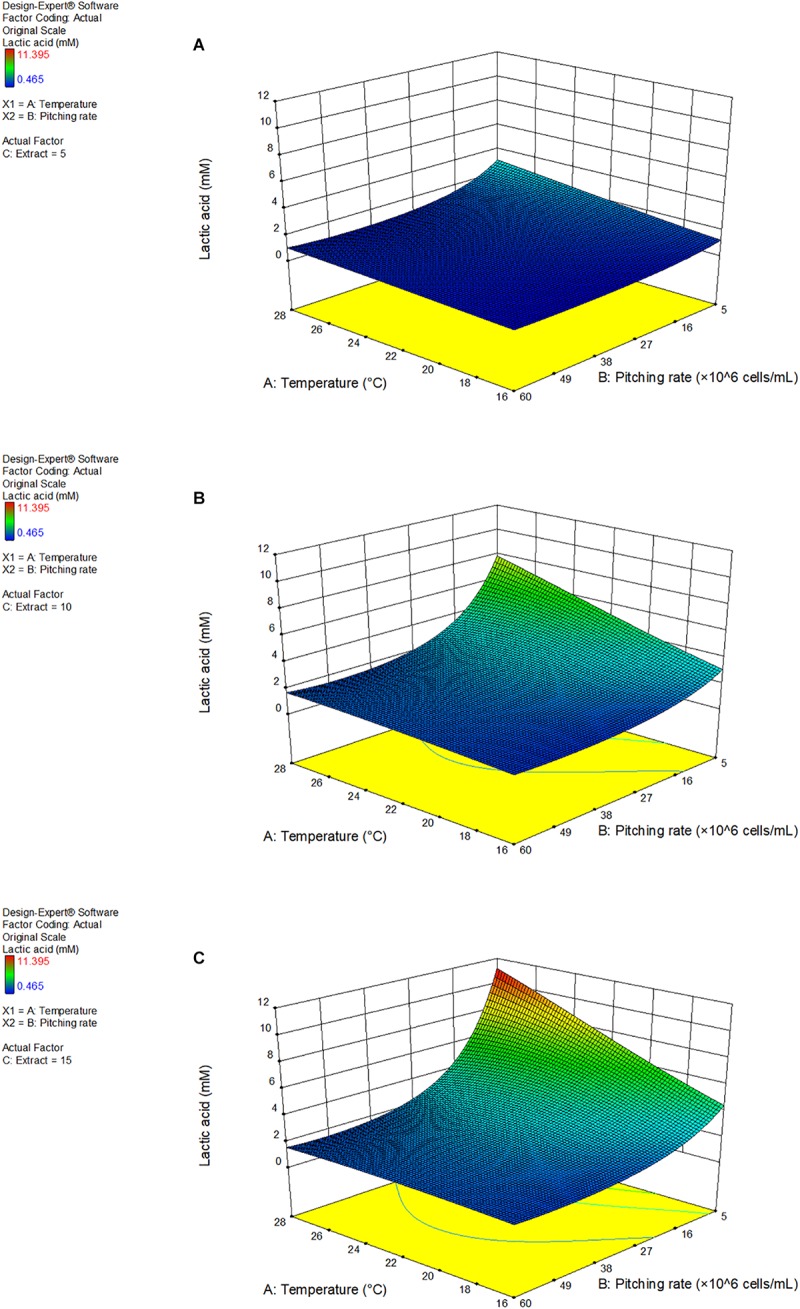
3D response surface model of response factor lactic acid as a function of fermentation temperature and pitching rate at 5°P **(A)**, 10°P **(B)**, and 15°P **(C)**. Model details and statistics in Supplementary Data Sheet S2.

Figure 4 shows the response surface as a 3D model of the lactic acid production at 5, 10, and 15°P, as a function of the fermentation temperature and pitching rate.

Increasing extract content enhanced the effect of the temperature and pitching rate parameters. Additionally, a low pitching rate had a very strong positive effect on the lactic acid production. Lactic acid also increased with an increasing fermentation temperature. The highest lactic acid concentration achieved was 11.4 mM at a pitching rate of 5 × 10^6^ cells/mL and a fermentation temperature of 28°C. A full table of the results of the response factors can be found in Supplementary Data Sheet S2.

##### Added glucose trial.

To investigate the hypothesis that lactic acid production can be boosted by the presence of higher amounts of glucose at the beginning of fermentation, a trial with a glucose-spiked wort sample was conducted. Table 7 shows the analytical results of the three worts used in this trial before and after fermentation with KBI 12.1.

**TABLE 7:** Analysis of worts before and after fermentation with KBI 12.1.

**Attribute**	**Unit**	**7°P**		**7°P +3% glucose**		**10°P**	
		**Wort**	**Fermented**	**Wort**	**Fermented**	**Wort**	**Fermented**
Attenuation	%	–	652^c^	–	470*a*	–	601*b*
App. extract	°P	7.350.01	2.570.12	9.660.02	5.080.03	9.990.01	3.950.09
Real extract	°P	7.350.01	3.500.08	9.660.02	5.980.02	9.990.01	5.150.08
Ethanol	% ABV	–	2.610.04*a*	–	2.590.02*a*	–	3.450.03*b*
pH		4.830.01	3.810.01^b^	4.880.01	3.460.09^a^	4.800.01	3.910.01^b^
FAN	mg/L	831	n.d.	837	n.d.	882	n.d.
Lactic acid	mM	–	5.190.11*a*	–	18.004.64*b*	–	5.100.26*a*
Fructose	g/L	1.280.01	<LOD	1.560.01	<LOD	2.090.01	<LOD
Glucose	g/L	6.050.01	<LOD	34.590.10	<LOD	8.520.05	<LOD
Sucrose	g/L	0.780.01	<LOD	0.780.00	<LOD	1.100.01	<LOD
Maltose	g/L	31.060.38	2.030.77	31.120.06	26.490.59	43.670.25	5.430.70
Maltotriose	g/L	9.050.11	9.900.08	9.030.02	8.460.13	12.700.02	13.560.11

*Values are given as means ± standard deviation. Different letters in superscripts indicate a significant difference between the fermented samples (p ≤ 0.05). n.d., not determined.*

**FIGURE 5:**
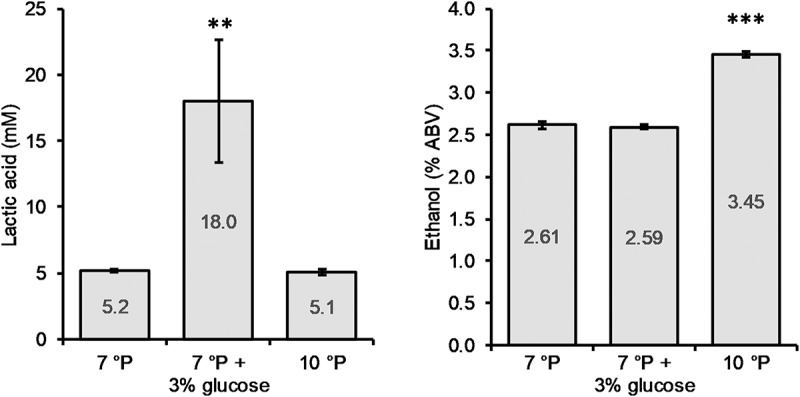
Final lactic acid and ethanol concentrations of 7°P wort extract, 7°P wort extract + 3% glucose, and 10°P wort extract after fermentation with KBI 12.1. **p ≤ 0.01; ***p ≤ 0.001.

The addition of glucose to the 7°P wort led to a significant increase in final lactic acid concentration (*p* < 0.01) of 246%, from 5.2 to 18.0 mM, while the final ethanol content of 2.6% ABV remained unchanged (Figure 5). The pH of the glucose spiked wort sample was correspondingly low, at 3.46. On the other hand, increasing the extract content from 7 to 10°P (without the addition of glucose) did not have an influence on the final lactic acid concentration (*p* > 0.05) but resulted in a significantly higher final ethanol content (*p* < 0.001) (Figure 5).

The monosaccharides fructose and glucose were depleted by the end of fermentation while maltose was never fully depleted (Table 7). Especially the fermented sample with spiked glucose, resulting in high lactic acid production, exhibited high residual maltose concentrations by the end of fermentation which is an indication for a premature inhibition by low pH and/or high lactic acid concentration.

### Pilot-Scale Brewing Trial

**FIGURE 6:**
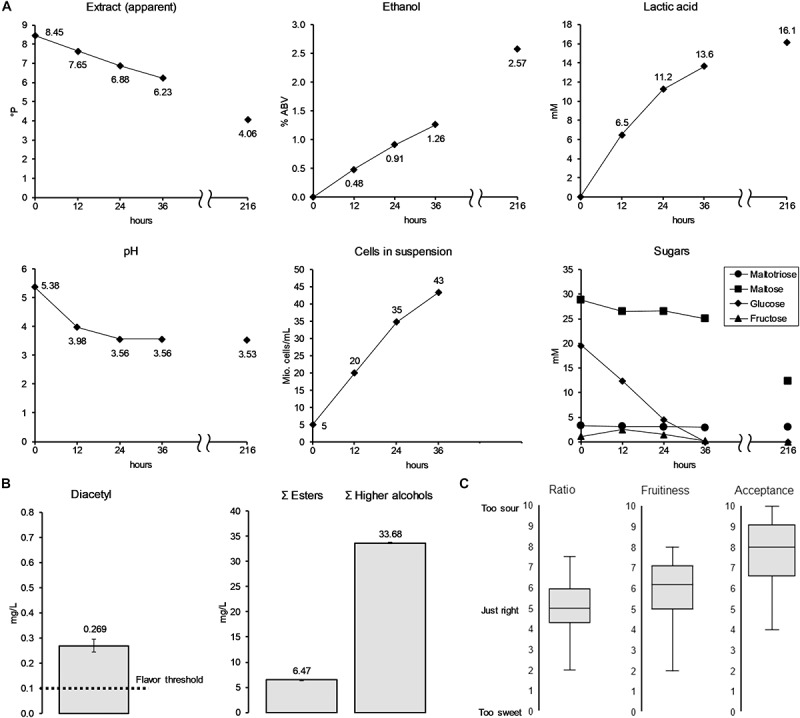
Analyses of pilot-scale (60 L) fermentation **(A)** of low alcoholic beer with KBI 12.1. Fermentation by-products **(B)** and sensory data **(C)** of the low alcoholic beer corresponds to the fermentation data at 36 h (finished low alcohol beer). Values at 216 h show the beer at final attenuation reached without the interuption of fermentation.

The results from the lactic acid optimization experiment gave valuable insights for the process development of a scaled-up brewing trial. The RSM results indicated that a low pitching rate and high fermentation temperature are favorable for increased lactic acid production, while the spiked glucose trial indicated that lactic acid production can be boosted by the initial glucose concentration of the wort. Considering these insights, amyloglucosidase was added during the mashing process of wort production to increase the amount of glucose relative to maltose. At the same time, a low pitching rate, at 5 × 10^6^ cells/mL, together with a high fermentation temperature (25°C) was chosen to increase lactic acid production on the process side. The aim was to create a low alcohol beer (LAB) by stopping the fermentation prematurely, at a point where the produced lactic acid is in balance with the sweetness of the residual wort sugars. For that reason, samples were taken every 12 h until the fermentation was stopped by filtering out the yeast by means of a plate filter. Figure 6 illustrates fermentation progress as well as results from volatile fermentation by-products analysis and sensory evaluation of the produced LAB (36 h).

The ethanol concentration of the beer at interruption of fermentation after 36 h had reached 1.26% ABV. The lactic acid concentration reached 13.6 mM (= 1.23 g/L) at a final pH of 3.56. Final apparent extract of the LAB was 6.23°P. The cell count showed a constant growth in the first 24 h, after which it slowed down to a cell concentration at time of filtration of 43 × 10^6^ cells/mL. Glucose was fully depleted after 36 h of fermentation while 0.17 mM (0.24 g/L) of fructose remained. Maltose only saw a small decrease and maltotriose was left untouched. The analysis of the beer that was left in the fermenter to reach final attenuation (216 h) showed only a small further increase in lactic acid to a concentration of 16.1 mM, while doubling in ethanol concentration to a final value of 2.57% ABV. At final attenuation, only about 55% of the maltose was consumed, with maltotriose concentrations unchanged. Analysis of volatile fermentation by-products of the stopped fermentation LAB revealed a low ester concentration of 6.5 mg/L (Figure 6B). At 0.27 mg/L, the diacetyl value was as well above its flavor threshold of 0.02–0.10 mg/L (Meilgaard, 1975; Saison et al., 2009). Diacetyl, an unwanted buttery flavor compound, is a fermentation by-product which, at the end of fermentation, is often at concentrations higher than its flavor threshold. In that case, a diacetyl rest is applied to allow yeast to reduce diacetyl to concentrations below the flavor threshold. In this study, the yeast was separated from the young beer before final attenuation was reached, and therefore reduction of the diacetyl concentration was not possible.

The results of the sensory evaluation indicated that a balanced ratio between residual sweetness from maltose and maltotriose and acidity from lactic acid was reached (Figure 6C). Fifty percent (interquartile range IQR; 50% of total reported values) of the panelists rated the sweetness/acidity ratio between 4.2 and 6.0 at a scale from 0 to ten, with 0 “too sweet,” 5 “just right,” and 10 “too sour.” Values corresponded to residual sugars of 17.0 mM (12.4 g/L) maltose, 6.2 mM (3.1 g/L) maltotriose, and 0.17 mM (0.24 g/L) fructose and a lactic acid concentration of 13.6 mM (1.23 g/L). The fruitiness was rated medium to high with the IQR ranging from 5–7 out of 10. Overall acceptability was rated with an IQR ranging from 6.5–9.0 out of 10.

## Discussion

In this study, we investigated four *Lachancea fermentati* strains isolated from kombucha. Genome analysis was performed to gain fundamental insights, to elucidate intraspecific differences due to their origin, and in an attempt to link the strains’ genotypes to their phenotype in wort fermentations. The strains were characterized by, e.g., their sugar utilization and stress sensitivities to evaluate their suitability in beer brewing. Screening in wort was performed to investigate intraspecific differences and to determine the best lactic acid producer. Subsequently, the fermentation parameters temperature, pitching rate, and glucose concentration were investigated to enhance lactic acid production. Finally, a low alcohol beer was produced at pilot-scale under optimized conditions.

The results from the genome analysis showed that the four kombucha isolates were diverse and generally separated into two groups, relating to their origin. The diploid isolates KBI 1.2, KBI 3.2, and KBI 12.1 exhibited high heterozygosity, an indication for intraspecific hybrids. Potentially, the isolates share a common ancestor based on patterns in LOH. This hypothesis is supported by the geographically close origin of KBI 1.2 and KBI 3.2, the United States. Due to the remote geographical origin of KBI 12.1 (Hawaii), its close phylogenetic relationship to KBI 1.2 and KBI 3.2, in contrast to KBI 5.3, calls for the assumption that an exchange of kombucha cultures between the Conterminous United States and Hawaii has taken place at some point. In fact, the exchange of kombucha cultures between kombucha brewers, and kombucha brewer communities has been common practice in the United States (The New York Times, 2010). Unlike the isolates from the United States, KBI 5.3, which originates from Australia, showed a closer phylogenetic relationship to CBS 6772, which originates from South Korea, and CBS 707, whose country of origin is unknown. Unfortunately, to date, very limited sequence data is available for comparison.

Generally, compared to the extensively studied species *Saccharomyces cerevisiae*, the species *L. fermentati* or even the *Lachancea* genus has not been investigated thoroughly. Consequently, only with the initial assumption of a strong degree of homology between the yeast species, assumptions about the *Lachancea fermentati* metabolism can be made.

The greater resistance to low pH conditions of the *Lachancea* strains, compared to the brewers’ yeast during the stress test, could tentatively be connected to their tendency to produce significant amounts of lactic acid during alcoholic fermentation. The strains must constantly export lactate and H^+^ out of the cell to maintain proton motive force and for this reason may be pre-adapted to high concentrations of H^+^-ions. In addition, the acidic kombucha environment has also likely selected for strains with enhanced tolerance to high acid concentrations and low pH values (Chakravorty et al., 2016).

Beside obtaining fundamental insights into the strains’ characteristics, we aimed to optimize the lactic acid production by *L. fermentati* during fermentation. The observed values of lactic acid production (between 1.33 and 3.47 mM) in wort extract of the investigated *L. fermentati* strains were low compared to previously reported values. Osburn et al. (2018) reported lactic acid production of 10 mM by a *L. fermentati* strain in a 11.4°P wort at a pitching rate of approximately 5 × 10^6^ cells/mL and 21.7°C. In a previous study with KBI 12.1, Bellut et al. (2019a) reported the production of 14.4 mM of lactic acid in a 6.6°P wort at a pitching rate of 8 × 10^6^ cells/mL and 25°C. However, the aforementioned studies used different fermentation conditions (e.g., pitching rate, temperature) and substrates, which has a significant influence on the lactic acid production, as we have shown in this study. Bellut et al. (2019b) already reported significant differences in lactic acid production by KBI 12.1 in different substrates from cereals, pseudocereals and pulses which could not be traced back to the sugar spectrum or free amino acid spectrum, further underlining the poor state of knowledge regarding factors that modulate lactic acid production in *Lachancea fermentati*.

Whole genome sequencing was performed to connect observations in the phenotype to the genotype of the individual strains. KBI 5.3 carried a mutation (397C > T) in the gene LAFE_0A07888G, resulting in a premature stop codon (Gln133^∗^) (Supplementary Data Sheet S1). LAFE_0A07888G is a gene with high similarity to the *JEN1* gene in *S. cerevisiae*. *JEN1* encodes for the monocarboxylate transporter Jen1 that was shown to be a lactic acid exporter (Casal et al., 1999), enhancing lactic acid yield in *S. cerevisiae* strains transformed with bacterial lactic acid dehydrogenases (Branduardi et al., 2006; Pacheco et al., 2012). The nonsense mutation in the *JEN1*-homolog of KBI 5.3 could tentatively have been the reason for the significantly low lactic acid production in comparison to the other *L. fermentati* strains. However, besides Jen1p, at least one other lactic acid transporter exists (Pacheco et al., 2012), which could tentatively explain the remaining, albeit low, lactic acid production. In addition, a single nucleotide deletion (230delT) was also observed in LAFE_0E15192G in the strain KBI 5.3 (Supplementary Data Sheet S1). LAFE_0E15192G shows some similarities with *S. cerevisiae* YML054C *CYB2*, a cytochrome b2 (L-lactate cytochrome-c oxidoreductase) component of the mitochondrial intermembrane space which is required for lactate utilization (and repressed by glucose and anaerobic conditions) (Sauer et al., 2010). This frameshift mutation could tentatively explain the inability to grow in lactic acid as the sole substrate in the API test. However, these effects should be tested in future studies by reverse engineering.

The RSM results have shown that in order to boost lactic acid production, a low pitching rate should be used in combination with a high fermentation temperature. Furthermore, in the favored conditions, a higher initial extract led to higher lactic acid concentrations. The fact that the samples with high lactic acid production did not reach final attenuation (Supplementary Data Sheet S2) was suggested to be caused by end-product inhibition through the mechanism described in section Stress Tests. Combined with the knowledge that glucose is commonly taken up at the beginning of fermentation before high amounts of maltose, sucrose or maltotriose are consumed (Horák, 2013), it was hypothesized that the increased lactic acid production in the worts with higher extract during the RSM trial was attributed to a higher amount of glucose. The results from the trial with spiked glucose indicated, that lactic acid production by KBI 12.1 can indeed be modulated by the amount of glucose present at the start of fermentation. Within the investigated range of 7–10°P, a higher amount of glucose resulted in increased lactic acid production, but without an increased ethanol production.

The RSM optimization and added glucose trial have shown that lactic acid production by KBI 12.1 is highly dependent on the pitching rate, fermentation temperature and initial glucose concentration. Lactic acid production by the strain KBI 12.1 varied from 0.5 to 18.0 mM based on the fermentation conditions and substrate composition. It was shown that, in order to increase lactic acid production by *Lachancea fermentati* KBI 12.1 in wort, the glucose concentration should be as high as possible, the pitching rate low (5 × 10^6^ cells/mL) and the fermentation temperature high (≥25°C).

The high lactic acid production in samples with a low pitching rate suggests that lactic acid production mostly took place during the growth phase at the beginning of fermentation. In contrast, under the same conditions, but at high pitching rates, little lactic acid was produced. This hypothesis was supported by fermentation data from the scaled-up brewing trial where 84% of total lactic acid was already produced in the first 36 h of fermentation, while the cells in suspension grew from 5 to 43 × 10^6^ cells/mL. However, in the case of the scaled-up fermentation, the lactic acid production also correlated with the consumption and depletion of glucose.

**FIGURE 7:**
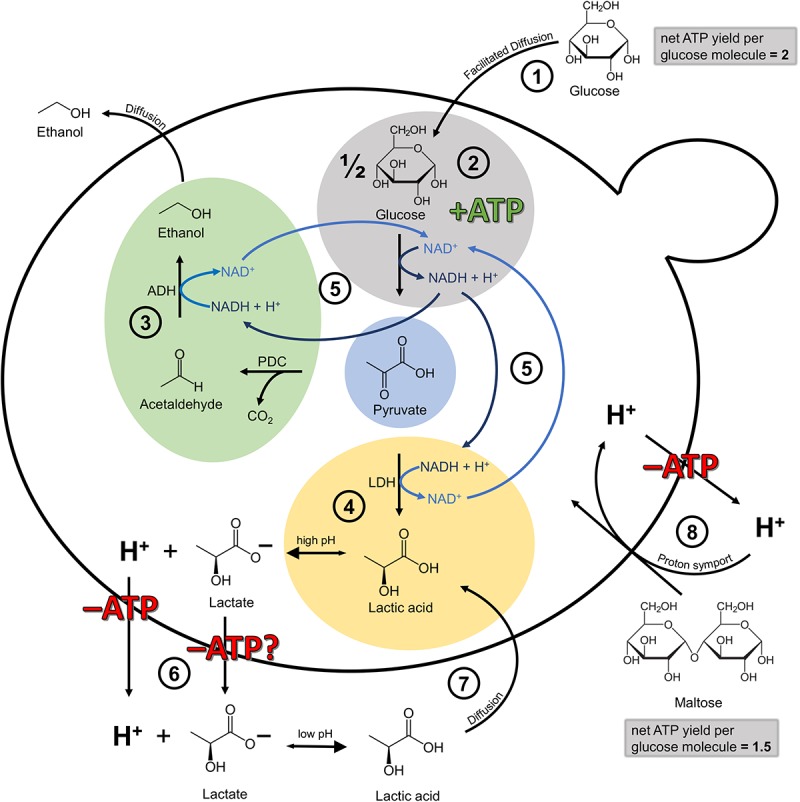
Simplified illustration of the cellular mechanisms involved in lactic acid production and self-inhibition in *Lachancea fermentati* in anaerobic wort fermentations under the assumption of fundamental homology to *Saccharomyces cerevisiae*. Adapted from Sauer et al. (2010) and Bellut et al. (2019a). **(1)** Glucose transport into the cell by facilitated diffusion. The net ATP yield per glucose molecule is 2. **(2)** Glycolysis, yielding one molecule of ATP per molecule pyruvate formed and one molecule of NADH which has to be recycled to NAD^+^. **(3)** Ethanol production via pyruvate decarboxylase (PDC) and alcohol dehydrogenase (ADH). The ethanol can leave the cell by passive diffusion. **(4)** Lactic acid production via lactic acid dehydrogenase (LDH). At high intracellular pH, the lactic acid dissociates into lactate and H^+^. **(5)** Both, ethanol formation and lactic acid formation are a means to recycle NADH to NAD^+^. **(6)** At the very least, the H^+^ has to be exported out of the cell at the expense of one molecule of ATP. In the worst case scenario, ATP-dependent mechanisms may be involved in both proton and anion export (Abbott et al., 2009; Mans et al., 2017). **(7)** At low extracellular pH, lactic acid is present in its protonated form and can enter the cell again via passive diffusion, creating an energy-requiring cycle with **6**. **(8)** Maltose transport into the cell is facilitated via proton symport. Consequently, the proton has to be exported out of the cell at the expense of ATP. For that reason, the net ATP yield per glucose molecule from maltose is 1.5 instead of 2.

On a molecular level, the metabolization of pyruvate via lactic acid dehydrogenase is an additional means of NADH recycling, with pyruvate decarboxylase and alcohol dehydrogenase being the more common pathway. NADH is produced during glycolysis, the yeasts’ ATP-generating pathway under anaerobic conditions, and has to be recycled to NAD^+^ (Figure 7). However, while ethanol can leave the cell by passive diffusion, lactic acid has to be actively transported out of the cell at the expense of ATP. This is due to the fact that at high intracellular pH, lactic acid dissociates into lactate and a proton. In order to maintain proton motive force and intracellular pH, this proton has to be exported via the plasma membrane H^+^-ATPase, with the expense of one ATP per proton. In the worst case, lactate export is also ATP-dependent, though the exact mechanisms are still unknown (Maris et al., 2004; Abbott et al., 2009; Mans et al., 2017). Abbott et al. (2009) confirmed that the lactic acid export requires energy in the form of ATP, which was shown by a full ATP depletion during anaerobic homolactate fermentation with a *S. cerevisiae* strain. Outside of the cell, at a low extracellular pH, the lactic acid is again present in its protonated form and can thus permeate the cell membrane via passive diffusion, creating an energy-requiring cycle (Sauer et al., 2010). Available evidence suggests therefore that the recycling of NADH via the lactic acid pathway seems to be more expensive for the cell than the ethanol pathway. Why the lactic acid pathway is chosen in the first place, at least at the beginning of fermentation, is still unknown and highlights the need for more research in this area. Presumably, the simultaneous recycling of NADH via the lactic acid and the ethanol pathway, resulting in an accumulation of lactic acid, developed as a strategy to compete with other microbes, comparable to the “make-accumulate-consume” strategy for ethanol in *S. cerevisiae* (Piškur et al., 2006; Pfeiffer and Morley, 2014). In a study on *S. cerevisiae*, Pacheco et al. (2012) found that when glucose is present, the produced lactic acid is exported out of the cell via Jen1 and Ady2, but when glucose (acting as the single carbon source) is depleted, the transporters are also actively involved in lactic acid consumption.

There is a general consensus that all maltose transport systems in *S. cerevisiae* so far characterized mediate the transport into the yeast cells against a concentration gradient in symport with protons. This proton import is balanced by proton export via the plasma membrane H^+^-ATPase, at the expense of one ATP per proton. This means, that the uptake of one molecule of maltose comes at the expense of one molecule of ATP (De Kok et al., 2012). Consequently, while glucose enters the cell via facilitated diffusion, maltose has to be actively imported into the cell via proton symport. The consequent export of the proton at the expense of ATP lowers the net ATP yield from maltose to 1.5 ATP per glucose molecule, instead of 2 ATP per molecule of glucose which entered the cell via facilitated diffusion. Figure 7 illustrates the simplified cellular mechanisms involved in lactic acid production and proton motive force maintenance in *Lachancea fermentati* in anaerobic wort fermentations assuming fundamental homology to *S. cerevisiae*.

The results from this study indicate that at the beginning of fermentation, at relatively high pH, low lactic acid concentration and the presence of glucose, *L. fermentati* KBI 12.1 can afford to simultaneously recycle NADH via the lactic acid pathway. A shift toward the ethanol pathway as the sole means of NADH recycling seemed to occur once glucose was depleted and the proton motive force maintenance became more costly due to a reduced net ATP yield from maltose compared to glucose, combined with an increasing stress caused by increased lactic acid concentrations (Figure 7). However, although our results give first indications on the underlying mechanisms for lactic acid production modulation in *L. fermentati* in wort fermentations, more research on ATP utilization and redox balance is necessary to draw conclusions.

It was possible to create a low alcohol beer (1.26% ABV) with KBI 12.1 by interrupting the fermentation after 36 h. The panelists evaluated the ratio of residual sweetness to acidity caused by lactic acid as balanced, giving a good indication for future applications. However, by removing the yeast from the wort prematurely, significant amounts of diacetyl were left in the young beer which can negatively affect the flavor of the beer, limiting the use of this strain for stopped fermentation. The high diacetyl concentration could potentially be tackled post-fermentation with an enzyme treatment by immobilized α-acetolactate decarboxylase (Krogerus and Gibson, 2013). In a previous study, Bellut et al. (2019a) produced a low alcohol beer with KBI 12.1 from a 6.6°P wort. However, the ethanol concentration was, at 2.6% ABV, considerably higher after final attenuation was reached, compared to 1.26% ABV after the interruption of fermentation in this study. Due to the consumption of all fermentable sugars and a high lactic acid production (14.4 mM), the taste of the beer was also characterized as sour. However, diacetyl was below its flavor threshold since the fermentation came to a halt naturally.

## Conclusion

To conclude, while the exact mechanisms for lactic acid production in *Lachancea fermentati* remain unknown, we have elucidated influencing factors and were able to shine some light on the KBI 12.1 strain’s behavior in wort fermentations regarding an enhanced lactic acid production and its consequent induction of a premature fermentation inhibition. We showed that the strain can afford the energy-expensive lactic acid production until a high concentration is reached (here up to 18 mM) only as long as glucose is present in the wort. A low alcohol beer could be produced which had a balanced profile between sweetness from residual sugars and acidity from the produced lactic acid. However, due to the premature cessation of fermentation, diacetyl was present above its flavor threshold. Future application trials should focus on finding the ideal extract value and ideal sugar spectrum of the wort to facilitate high lactic acid concentrations in balance with residual sweetness while still reaching final attenuation. To validate the hypothesis of the influence of the mutated *JEN1-* and *CYB2-*similar genes in KBI 5.3 leading to a reduced lactic acid production, gene knock-out experiments in the strains without the mutation could lead to further insights regarding the modulation of lactic acid production in *Lachancea fermentati*.

## Data Availability Statement

The datasets generated for this study can be found in the Illumina reads generated in this study have been submitted to NCBI-SRA under BioProject number PRJNA587400 in the NCBI BioProject database.

## Ethics Statement

Ethical approval was required for this study in accordance with local guidelines. The sensory trial was carried out in accordance with IFST Guidelines for Ethical and Professional Practices for the Sensory Analysis of Foods and the panel provided written informed consent to take part in the research.

## Author Contributions

KB performed the fermentation experiments, analyses, and statistics described in this study. KK performed flow cytometry, and bioinformatic analysis of the whole genome sequence data. KB, KK, and EA designed the experiments. KB and KK wrote the manuscript. EA supervised this study. All authors read and approved the final manuscript.

## Conflict of Interest

KK was employed by VTT Technical Research Centre of Finland Ltd.

The remaining authors declare that the research was conducted in the absence of any commercial or financial relationships that could be construed as a potential conflict of interest.

The handling editor declared a past co-authorship with one of the authors KK.

## Footnotes

1 http://gryc.inra.fr

2 https://blast.ncbi.nlm.nih.gov/Blast.cgi
