# Supplementary figures and images for: Lachancea fermentati Strains Isolated From Kombucha: Fundamental Insights, and Practical Application in Low Alcohol Beer Brewing
Source: Front Microbiol. 2020 Apr 23;11:764. doi: 10.3389/fmicb.2020.00764 (PMC7191199; doi:10.3389/fmicb.2020.00764)

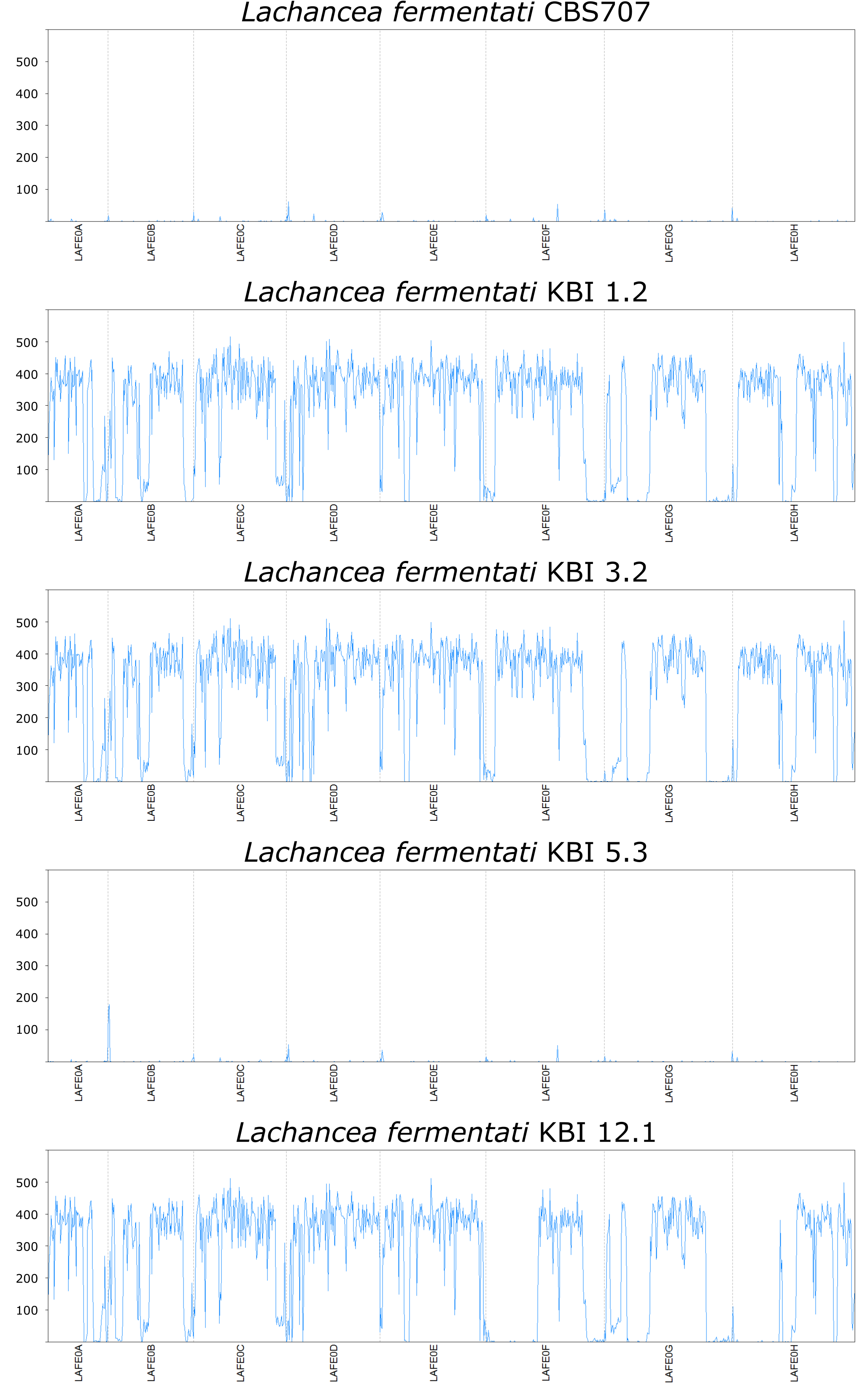

Supplement: Supplementary file 1 [file Image_1.PNG]

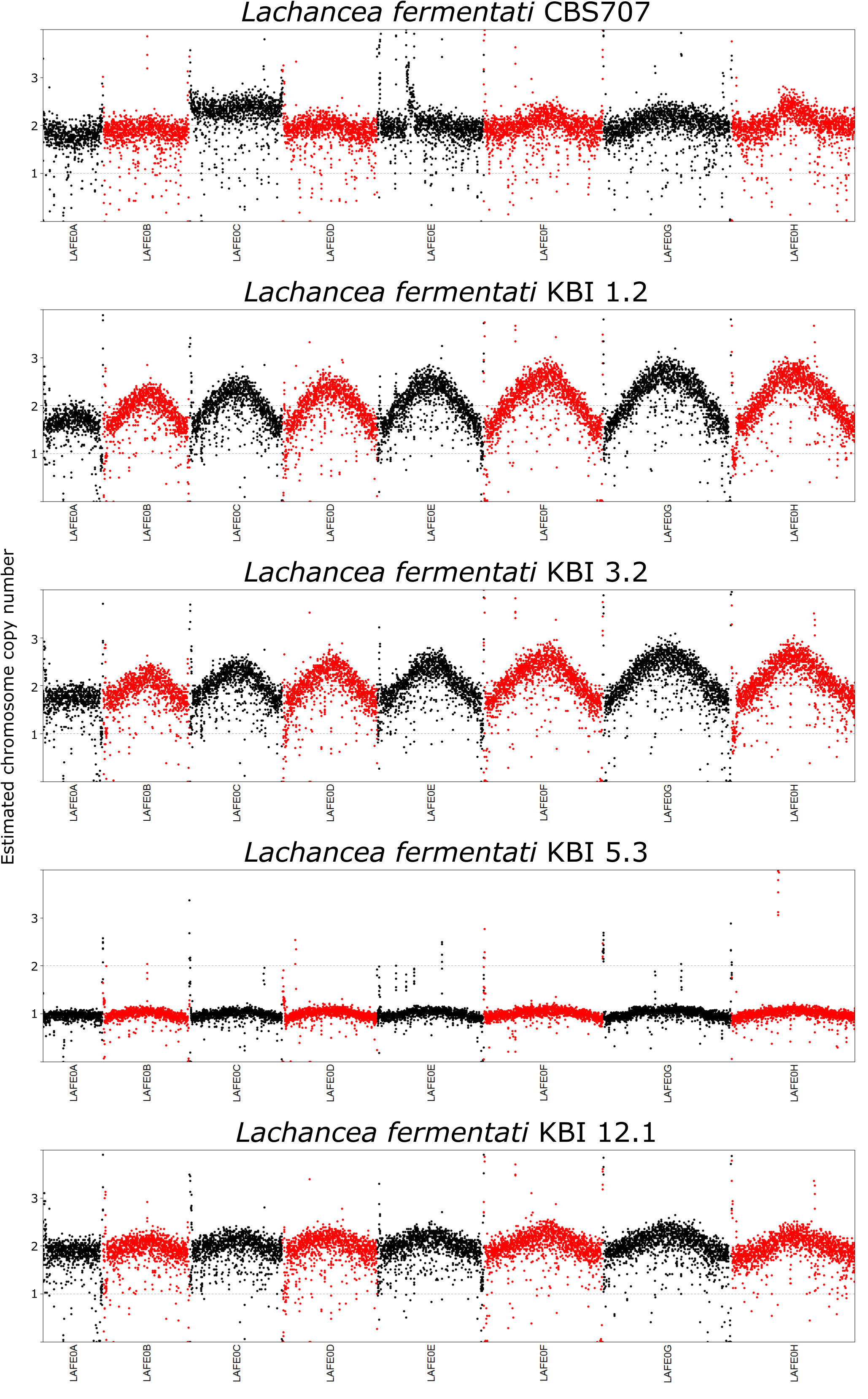

Supplement: Supplementary file 2 [file Image_2.PNG]

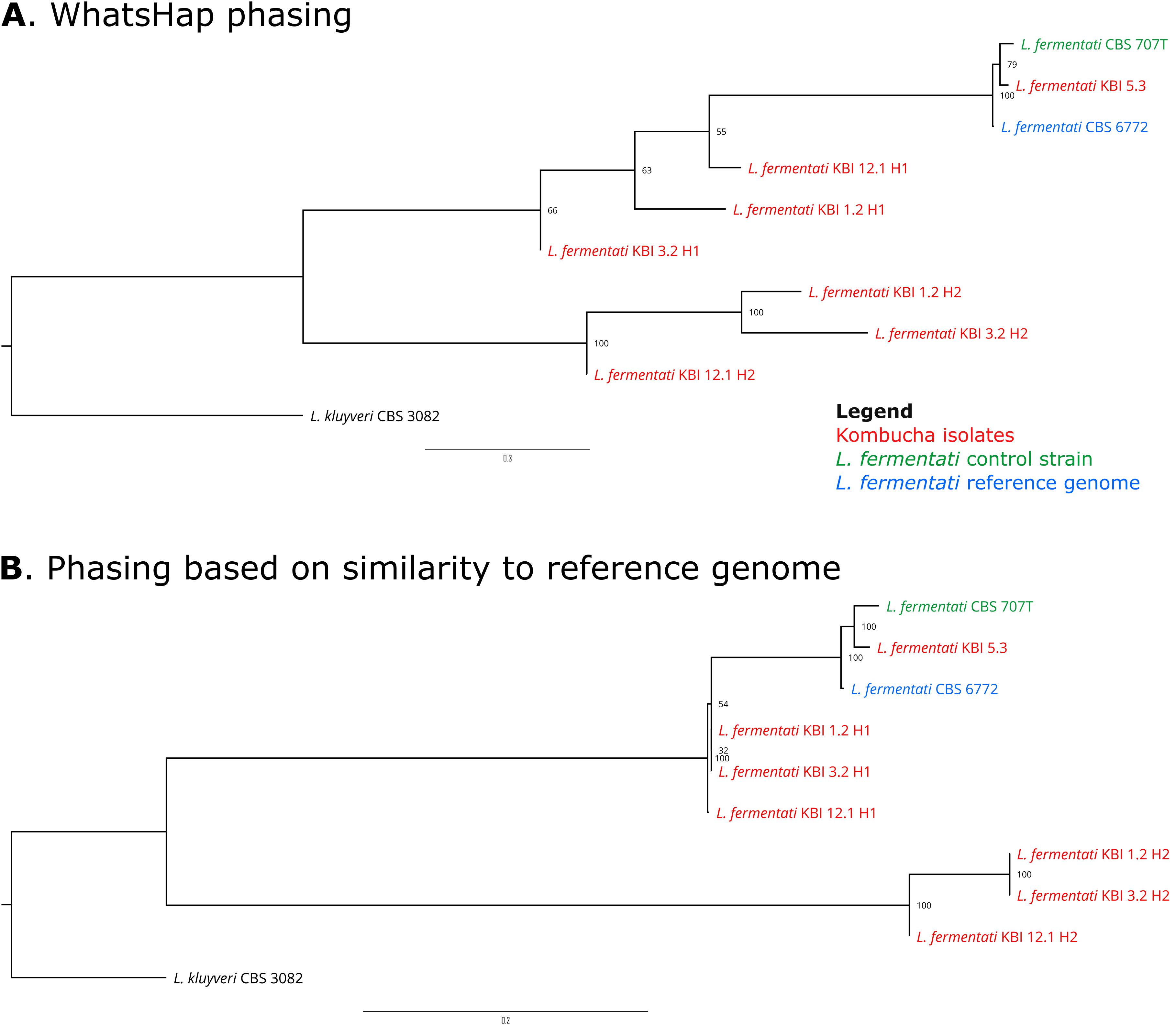

Supplement: Supplementary file 3 [file Image_3.PNG]

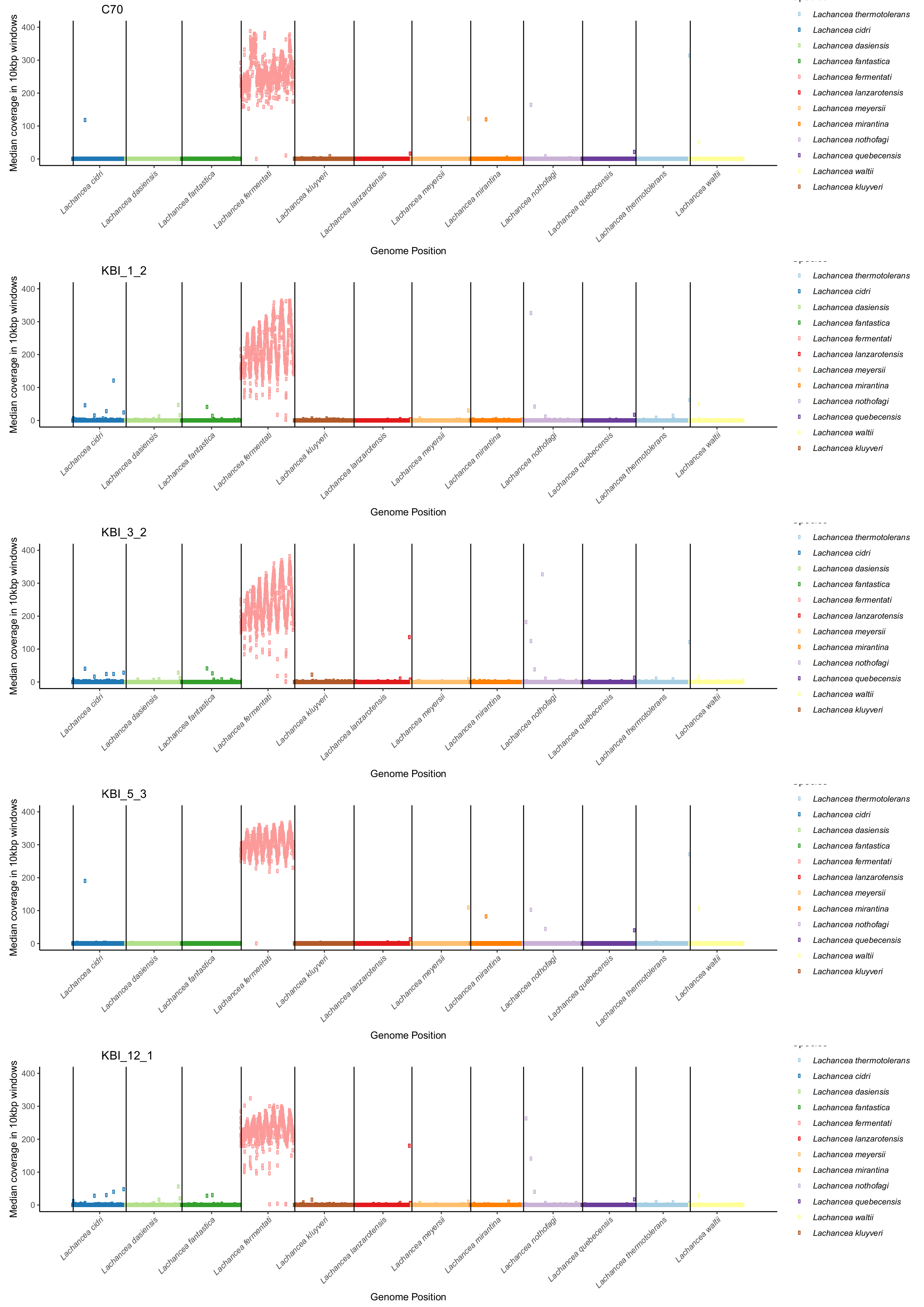

Supplement: Supplementary file 4 [file Image_4.PNG]
