# Supplementary material for: Lachancea fermentati Strains Isolated From Kombucha: Fundamental Insights, and Practical Application in Low Alcohol Beer Brewing
Source: Front Microbiol. 2020 Apr 23;11:764. doi: 10.3389/fmicb.2020.00764 (PMC7191199; doi:10.3389/fmicb.2020.00764)
Supplement: Supplementary file 7 [file Table_3.docx]

Supplementary Figure 1. The number of heterozygous single nucleotide polymorphisms (in 10 kbp windows) in the five sequenced *Lachancea fermentati* strains compared to the *L. fermentati* CBS 6772 (NCBI Accession GCA_900074765.1) reference genome. Values close to zero indicate regions lacking heterozygosity.

Supplementary Figure 2. Estimated chromosome copy numbers of the five sequenced *Lachancea fermentati* strains based on the sequencing coverage (median coverage in 1 kbp windows) of reads aligned to the *L. fermentati* CBS 6772 (NCBI Accession GCA_900074765.1) reference genome.

Supplementary Figure 3. Maximum likelihood phylogenetic trees based on phased single nucleotide polymorphisms (SNPs) at 6330 sites in the six *L. fermentati* and one *L. kluyveri* genomes (rooted with *L. kluyveri* as outgroup). Numbers at nodes indicate bootstrap support values. Branch lengths represent the number of substitutions per site. SNPs in (A) were phased with WhatsHap based on reads containing two or more heterozygous SNPs, while SNPs in (B) were phased based on similarity to the reference genome as described in Ortiz-Merino et al. (2018).

Supplementary Figure 4. The median coverage in 10 kbp windows of sequencing reads from the five sequenced *Lachancea fermentati* strains aligned to a concatenated reference genome consisting of 12 species in the *Lachancea* genus. Reads align exclusively to *L. fermentati*, ruling out that any of the strains were interspecific hybrids.

Supplementary Data Sheet S1. Tab 1) Full data sets of API substrate test of the respective strains. Tab 2) Relative amounts of volatile analysis of the fermented worts during screening. Tab 3) Quantitative amounts of volatile analysis of the fermented worts during screening. Tab 4) List of high-impact mutations. High-impact mutations discussed are highlighted yellow.

**Supplementary Data Sheet S2.** Detailed Response Surface Methodology (RSM) data and statistical analysis including model design, analysis of variance (ANOVA), and model diagnostics.
